# Overcoming Material Incompatibility via 2D Free‐Surface Engineering

**DOI:** 10.1002/adma.202505101

**Published:** 2025-08-11

**Authors:** Youcef A. Bioud, Meriem Bouchilaoun, Waldemar Schreiber, Redouane Amrar, Gilles Patriarche, Tao Ma, Jens Ohlmann, Ali Soltani, David Lackner, Stefan Janz

**Affiliations:** ^1^ Division Photovoltaics Fraunhofer Institute for Solar Energy Systems (ISE) Heidenhofstraße 2 79110 Freiburg Germany; ^2^ Department of Electrical Engineering Université de Sherbrooke Sherbrooke Québec J1K 2R1 Canada; ^3^ Laboratoire Nanotechnologies Nanosystèmes (LN2) ‐ CNRS UMI‐3463 Institut Interdisciplinaire d'Innovation Technologique (3IT) Université de Sherbrooke 3000 Boulevard Université Sherbrooke QC J1K OA5 Canada; ^4^ Université Paris‐Saclay, CNRS Centre de Nanosciences et de Nanotechnologies Palaiseau Paris 91120 France; ^5^ Michigan Center for Materials Characterization University of Michigan Ann Arbor Michigan 48109 USA

**Keywords:** 2D free‐surface engineering, defect‐free growth, epitaxy, material compatibility

## Abstract

Heteroepitaxy has been pivotal in advancing both optoelectronics and microelectronics, driving the development of faster, more efficient devices across diverse applications. However, achieving high material quality remains challenging due to lattice mismatches. Strain induced by variations in lattice parameters and thermal properties provides additional degrees of freedom for material tailoring but often leads to dislocation generation, wafer bowing, and cracking. These issues are addressed through a scalable post‐epitaxial approach that strategically targets the misfit dislocation network, leading to the creation of a sub‐nanometric 2D free surface (2DFS). This interface effectively decouples the epilayer from the substrate, significantly reducing strain‐related defects. Scalable heterostructures exhibited pronounced defect annihilation, as demonstrated by electron microscopy, defect etching, and photoluminescence analysis—an effect attributed to the surrounding free surfaces. This method strikes an optimal balance between bulk‐quality characteristics and high surface integrity, offering a new paradigm for achieving heteroepitaxial bulk‐class materials.

## Graphical abstract

A scalable post‐epitaxial strategy introduces a sub‐nanometric 2D free surface (2DFS) at the heterointerface, effectively decoupling lattice‐mismatched layers. This process significantly reduces strain‐driven defects and dislocation density in Ge/Si heterostructures. The resulting bulk‐like material quality, validated by advanced microscopy and optical analyses, opens new pathways for high‐performance heteroepitaxy across micro‐ and optoelectronic applications.

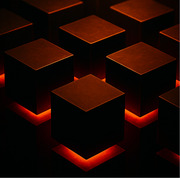

## 1 Introduction

The integration of heterogeneous materials remains a pivotal scientific and technological challenge. Successfully overcoming this hurdle promises to catalyze profound advancements across diverse fields, notably through the optimization of device performance by combining materials with disparate electronic and optical characteristics. This breakthrough has the potential to revolutionize quantum technologies,^[^ ^1^ ^]^ solar energy systems,^[^ ^2^, ^3^ ^]^ advanced imaging,^[^ ^4^ ^]^ light sources,^[^ ^5^ ^]^ memories^[^ ^6^ ^]^ and sensing applications,^[^ ^7^ ^]^ with far‐reaching impacts on artificial intelligence, data storage, automotive, healthcare, Internet of things and climate monitoring.^[^ ^8^, ^9^, ^10^, ^11^, ^12^, ^13^ ^]^ Moreover, achieving such integration through scalable methods could significantly democratize these technologies by reducing the high costs associated with current materials and complex fabrication processes. Despite remarkable progress in leveraging mismatched hetero‐epitaxial semiconductors, monolithic integration of high‐quality mismatched semiconductors remains a persistent challenge. This arises from the significant differences in lattice constants and thermal expansion coefficients between epitaxial layers and their hetero substrates.^[^ ^14^ ^]^ During growth, the induced strain in the epitaxial layer can either be stored as elastic strain energy or relieved through the formation of misfit dislocations at the interface, once a critical thickness is surpassed.^[^ ^15^ ^]^ This process is unfortunately accompanied by a high density of threading dislocations (TDs) extending toward the surface.^[^ ^16^ ^]^ TDs are particularly detrimental as they infiltrate active device regions, serving as non‐radiative recombination centers that compromise carrier mobility,^[^ ^17^ ^]^ lifetime,^[^ ^18^ ^]^ optical birefringence,^[^ ^19^ ^]^ and increase current leakage,^[^ ^20^ ^]^ thereby severely impairing device functionality. To address the persistent challenge of TDs in heteroepitaxial materials and meet industry standards, various strategies employing conventional substrates have been explored over recent decades. Approaches such as compositional grading,^[^ ^21^ ^]^ epitaxial lateral overgrowth,^[^ ^22^ ^]^ and selective area deposition^[^ ^23^ ^]^ have been investigated to mitigate TD densities. Despite their feasibility, these techniques often remain constrained to small‐scale applications and involve costly, complex processing technologies. Furthermore, the application of thick graded buffers can cause significant wafer bowing, making the epi‐wafers unsuitable for large‐scale fabrication with standard lithography and processing techniques.^[^ ^24^ ^]^

Recent advancements in heteroepitaxy have led to three innovative techniques: pinhole‐based epitaxy, remote epitaxy, and van der Waals epitaxy.^[^ ^25^ ^]^ Pinhole‐based epitaxy involves direct nucleation through pinholes in a 2D layer with lateral overgrowth,^[^ ^26^, ^27^ ^]^ while remote epitaxy relies on substrate bonding, ionicity, and polarity through ultrathin 2D van der Waals interlayers.^[^ ^28^, ^29^, ^30^, ^31^ ^]^ Van der Waals epitaxy utilizes van der Waals forces from the substrate surface.^[^ ^32^, ^33^, ^34^ ^]^ Despite their advantages, such as reduced defect formation, the industrial application of 2D materials continues to face challenges stemming from scalability limitations.^[^ ^35^, ^36^, ^37^, ^38^ ^]^

In contrast, Aspect Ratio Trapping offers a promising alternative for dislocation annihilation.^[^ ^39^, ^40^, ^41^ ^]^ This technique employs substrate patterning to terminate dislocations at the free surfaces of patterned SiGe/Si structures, potentially confining or eliminating them during the growth. When combined with compliant effects from porous Si pillars, Aspect Ratio Trapping shows potential for further reducing dislocations within the central regions of the pillars.^[^ ^42^ ^]^ However, scalability issues persist, with microstructures typically limited to smaller dimensions of ≈2 × 2 µm^2^, and high‐temperature growth conditions can adversely affect pore structures and thereby the compliance effect. Moreover, the rough surface quality associated with bottom–up approaches on unconventional substrates necessitates additional planarization steps for effective growth of active materials, such as III–V semiconductors. Our previous work has demonstrated a defect‐reducing strategy through top‐down methods, by tuning the motion of dislocations utilizing threading dislocation‐selective deep etching followed by thermal annealing to create nanovoids in the Ge layer that attract and annihilate dislocations.^[^ ^43^ ^]^ The averaged dislocation density is reduced by over three orders of magnitude, from ≈10^8^ cm^−2^ to a lower‐limit of ≈10^4^ cm^−2^ for micron‐thick Ge/Si layers.^[^ ^44^, ^45^ ^]^ Although this approach is effective, it necessitates additional polishing steps due to the high roughness induced by the direct electrochemical etching process on the surface. This requirement significantly increases integration costs, posing challenges for large‐scale industrial applications.^[^ ^46^ ^]^ This underscores the critical balance between the bulk quality of the epitaxial layer and its surface quality for effective device integration.

Here, we propose a novel technique designed to achieve an optimal balance between bulk‐quality attributes and high surface quality for mismatched systems. Ge/Si heteroepitaxy is chosen to demonstrate the concept, given its broad application potential in optoelectronics. This method aims to achieve a defect‐free epitaxial layer by precisely addressing the root cause of threading dislocations localized at the heterostructure interface—the misfit dislocation network—while preserving the surface integrity. By optimizing the doping profile, the process strategically directs electrochemical etching at the misfit dislocation network, thereby creating a sub‐nanometric, 2D free surface (2DFS). This 2DFS effectively decouples the Ge layer from the underlying Si substrate, enabling efficient strain relaxation and significantly reducing strain‐related defects typically observed in planar heterostructures.

This approach leverages standard industrial processes, such as dry and electrochemical etching, with minimal expected impact on overall device production costs. Notable benefits include the adaptability of active surface areas to specific applications and cost‐efficient implementation. Furthermore, this technique aligns with large‐scale semiconductor manufacturing, where both etching techniques are scalable and easily integrated into fully automated systems. Additionally, it relies on common materials and standard processing durations, utilizing existing, widely adopted production technologies.

Leveraging a scalable silicon platform, the 2DFS strategy enables the monolithic integration of lattice‐mismatched Ge/Si structures into CMOS circuits, addressing longstanding performance bottlenecks. Using this approach, Ge layers grown on Si exhibit enhanced structural and optical quality, making them suitable for electro‐absorption modulators,^[^ ^47^, ^48^ ^]^ as well as for high‐performance Ge/Si single‐photon avalanche diodes^[^ ^49^, ^50^ ^]^ and photodetectors^[^ ^51^, ^52^ ^]^ that could rival state‐of‐the‐art III–V devices. The sub‐nanometric separation interface further unlocks advanced processing routes, including layer transfer and kerfless wafering—key to the scalable fabrication of virtual Ge substrates.^[^ ^53^ ^]^ The platform also supports the formation of suspended Ge membranes on patterned Si,^[^ ^54^ ^]^ selective III–V regrowth on Ge‐buffered Si,^[^ ^55^ ^]^ and undercut laser cavities leveraging strain relaxation.^[^ ^56^ ^]^ Additionally, defect‐suppressed Ge layers provide a robust foundation for Ge‐rich Si_1−x_Ge_x_ waveguides, enabling low‐loss photonic interconnects on bulk Si.^[^ ^57^ ^]^ Replacing bulk Ge with thin Ge buffer layers on Si thus offers a compelling route toward cost‐effective, scalable multijunction solar cells for micro‐concentrated photovoltaics.^[^ ^53^, ^58^, ^59^, ^60^ ^]^

This work demonstrates that dislocation removal using a sub‐nanometer, 2DFS in Ge/Si heteroepitaxy offers a promising and innovative approach for defect annihilation in mismatched structures. At the core of this approach is the concept of “germanium on quasi nothing”, wherein the formation of a 2DFS precisely at the Ge/Si interface relieves substrate‐induced strain on the epitaxial layer. This atomic detachment facilitates the correction of existing defects through dislocation recombination or annihilation at the free surface surrounding the structure using a soft corrective annealing. Additionally, by avoiding the formation of continuous layers, this method mitigates wafer bowing and crack formation, further enhancing the structural integrity of the material.

Our findings confirm the viability of this method at an adequate area for active device integration, with promising scalability to larger dimensions for broader applications. The resulting structure can be seamlessly converted into a continuous 2D Ge layer, suitable for device fabrication, by strategically increasing the deposited thickness.^[^ ^54^ ^]^ Measurements of etch pit density (EPD) and transmission electron microscopy (TEM) observations indicate a significant reduction in dislocation density within the Ge layer of the 2DFS sample. Geometrical phase analysis (GPA) reveals a full strain relaxation of the epitaxial Ge layer, confirming its independence from the Si substrate. Additionally, hyperspectral photoluminescence (PL) measurements reveal a strong and stable PL emission from the Ge layer on the 2DFS substrate, even as the dimensions increase, in contrast to conventional Ge/Si layers. This breakthrough underscores the 2DFS approach's capability to produce epitaxial structures with bulk‐like properties, effectively addressing the challenges posed by lattice and thermal mismatch and establishing a new paradigm in semiconductor integration.

## 2 Results and Discussion

**Figure 1:**
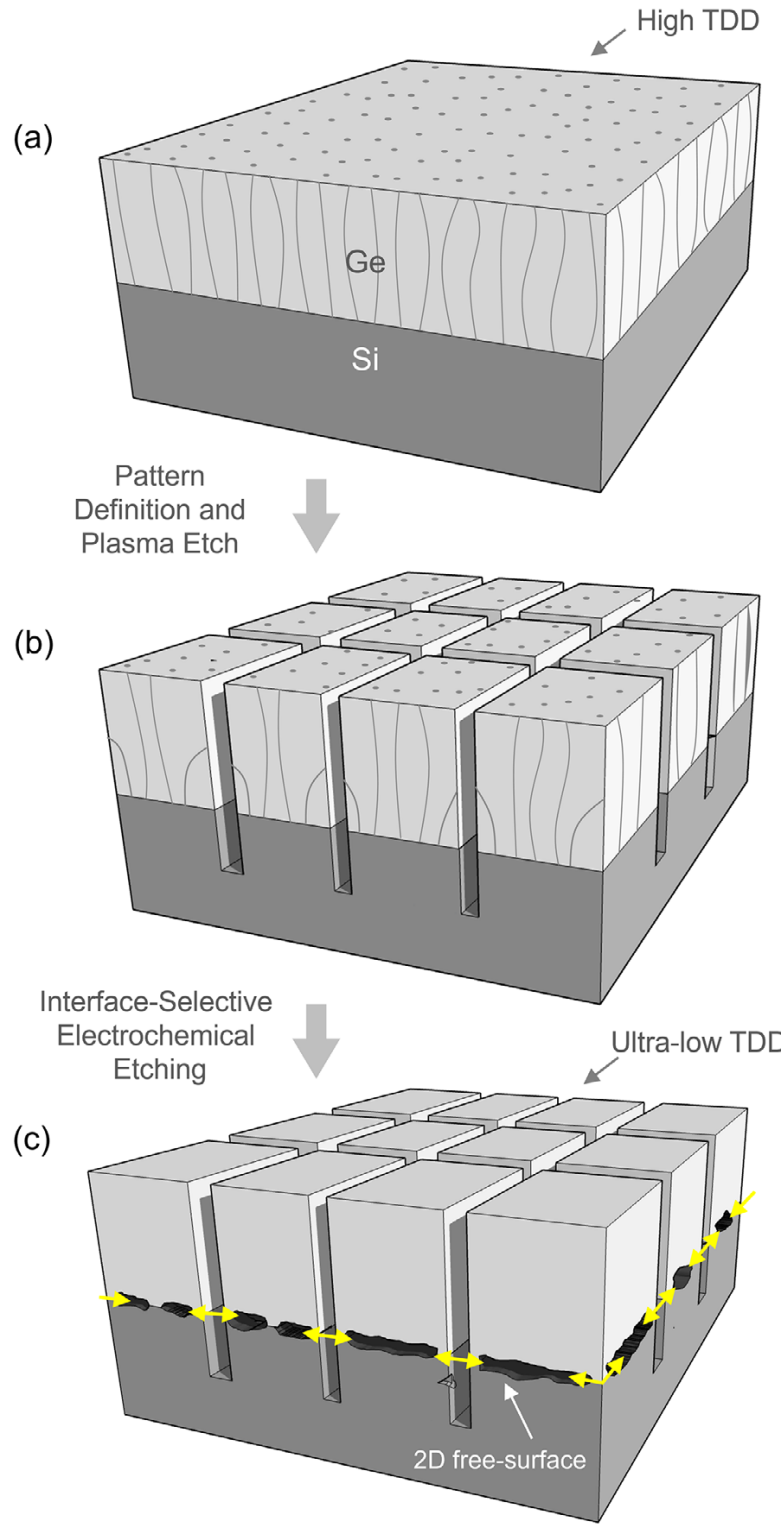
Schematic illustration of the Interfacial 2D Free‐Surface (2DFS) approach. a) Heteroepitaxial growth of a bulk Ge film on a (001) Si substrate, resulting in a high density of threading dislocations due to lattice mismatch. b) Formation of exposed sidewalls via Deep Reactive‐Ion Etching (DRIE), followed by cyclic annealing. This step promotes the bending and termination of threading dislocations at the vertical facets of the square‐shaped Ge/Si structure. c) Introduction of an interfacial 2D free surface by selective electrochemical etching along misfit dislocations at the Ge/Si interface (etch direction indicated by the yellow arrow). This configuration enables enhanced strain relaxation. Subsequent soft annealing facilitates dislocation glide and rearrangement, leading to the formation of isolated Ge microcrystals with substantially reduced dislocation density and near‐bulk crystalline quality. This engineered platform defines the 2DFS substrate.

In this study, a Ge/Si substrate undergoes a two‐step post‐epitaxial treatment aimed first at forming square‐shaped Ge/Si mesas with exposed sidewalls, followed by the generation of an interfacial 2D free surface, as schematically illustrated in **Figure** **1**a–c. The initial step involves plasma etching of the Ge/Si structure using the Bosch process, which provides access to the Ge/Si interface. The subsequent step entails selective electrochemical etching of the misfit dislocation network to produce a sub‐nanometric free surface that separates the Ge layer from the Si substrate. Following this, a soft annealing process promotes the migration and rearrangement of TDs, effectively eliminating them and resulting in the formation of Ge microcrystals with bulk‐quality attributes, both in surface and volume.

**Figure 2:**
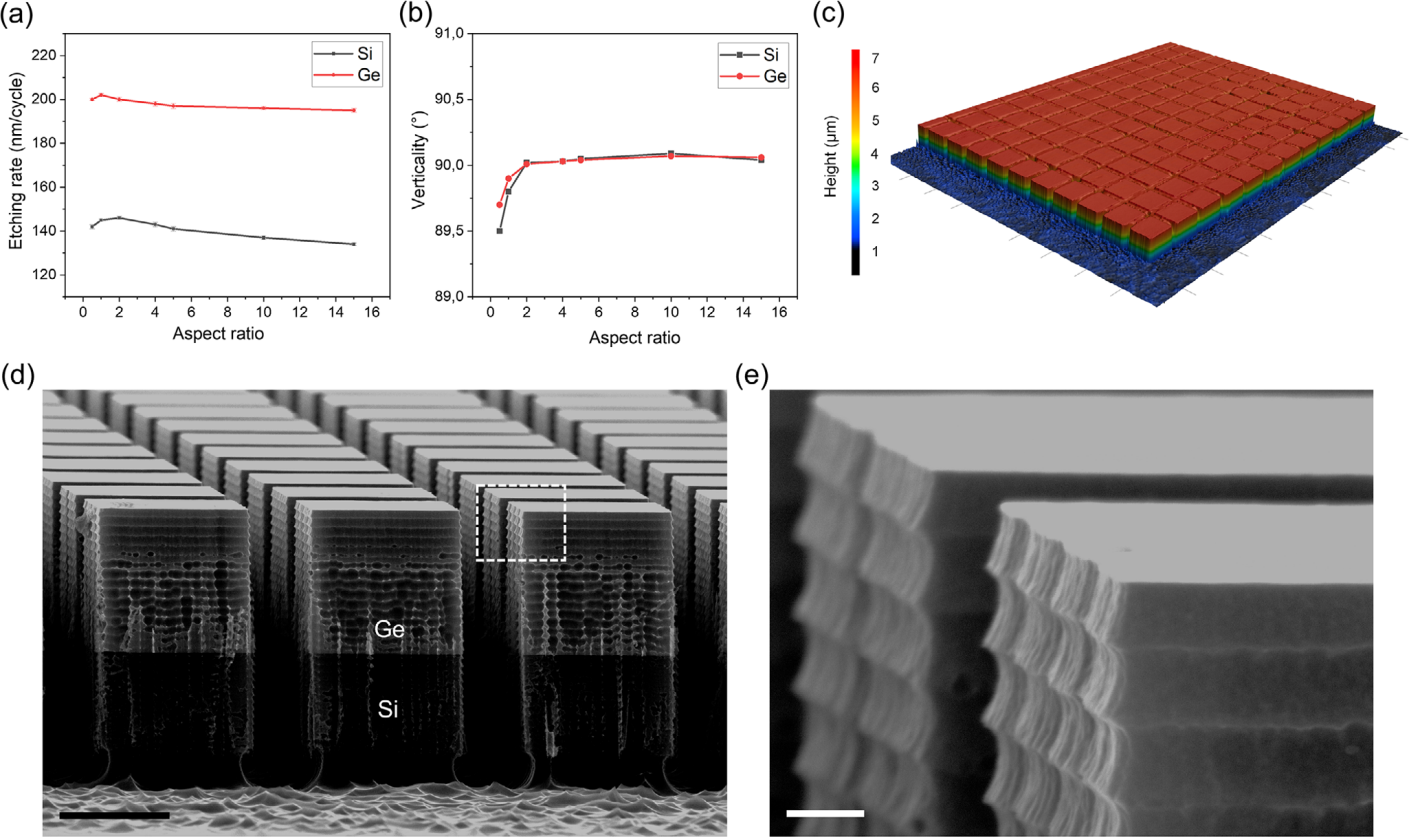
Fabrication of exposed sidewalls in the Ge/Si structure. a,b) Etch rate and profile verticality as a function of aspect ratio for both Ge and Si using the Bosch process, confirming high etch efficiency and precise anisotropic control. c) 3D optical microscopy mapping over a large area, demonstrating the uniformity of the patterned square‐shaped Ge/Si structures. d) SEM image of a 3 × 3 µm^2^ array of square‐shaped Ge/Si mesas. e) High‐resolution SEM inset from d) showing consistent Ge scallop morphology along the vertical sidewalls. Vertical etching is a key enabler for the subsequent creation of an interfacial 2D free surface at the Ge/Si interface via selective electrochemical etching. This process targets misfit dislocations while preserving the Ge surface integrity. Scale bars: 2 µm (d) and 200 nm (e).

For this study, 3 µm‐thick Ge layers on Si substrates with a high dislocation density (10^8^ cm^−2^) were patterned using electro lithography and deep reactive ion etching based on the Bosch process.^[^ ^61^ ^]^ A common challenge in plasma etching, particularly for high‐aspect‐ratio trenches, is the decreasing etch rate as the aspect ratio increases, as well as challenges in maintaining sidewall verticality.^[^ ^62^ ^]^ To address this, we investigated the etch rate and verticality as a function of aspect ratio for both Ge and Si, utilizing a gas mixture of SF_6_, C_4_F_8_, and O_2_. The effect of O_2_ content in SF_6_‐O_2_ gas mixtures on etch rate and sidewall profiles for Ge and Si in reactive ion etching has been previously documented.^[^ ^63^ ^]^ Ge typically etches faster than Si in conventional halogen plasmas at low bombardment energy.^[^ ^64^ ^]^ High etch rates were observed, ≈200 nm per cycle for Ge and 140 nm per cycle for Si, with minor variations depending on the aspect ratio, but remaining relatively stable for both materials (**Figure** **2**a). An O_2_ concentration of 10% resulted in optimal anisotropic mesa etching for both Ge and Si, producing nearly vertical sidewalls at 90° for an aspect ratio up to 2, as shown in Figure 2b. The average aspect ratio was calculated using the formula provided in Note S1 (Supporting Information).

To create the square‐shaped Ge/Si mesas with exposed sidewalls, we applied this etching protocol to trenches 1 µm wide and 6 µm deep, equally divided between the Ge and Si layers (3 µm each). After etching, a cleaning step using pure SF₆ plasma was performed to minimize residual micro‐masking. 3D optical microscopy across large areas (Figure 2c) demonstrated the uniformity of the square‐shaped Ge/Si structures. Figure 2d shows a scanning electron microscope (SEM) image of a 3 × 3 µm^2^ array of these structures, with the inset in Figure 2e providing a high‐resolution SEM image highlighting the uniform Ge scallop sizes.

During the initial stage of etching, Ge was isotropically etched, resulting in a rounded profile. In subsequent cycles, the passivation layer effectively protected the upper portion of the sidewalls, allowing etching depth to increase while forming characteristic scalloped profiles—commonly seen during time‐multiplexed Si etching.^[^ ^65^ ^]^ Upon reaching the Si layer, the scallop size decreased as the etch rate reduced. The final isotropic profile observed at the base of the towers is attributed to the cleaning step in pure SF₆. This etching protocol enabled the formation of uniform square‐shaped Ge/Si mesas with well‐defined sidewalls. These sidewalls offer crucial access to the Ge/Si interface for subsequent processing. However, their ability to support dislocation elimination is primarily effective in the vicinity of the mesa edges, as demonstrated in the bottom‐up approach.^[^ ^40^ ^]^ To further evaluate the potential of discontinuous epi‐layers obtained through a top‐down approach for dislocation elimination, we implemented a conventional cyclic annealing treatment.^[^ ^66^, ^67^ ^]^ The thermal stress generated during this process is expected to promote dislocation movement toward the nearest free surfaces, enabling dislocation bending at the sidewalls or self‐annihilation under favorable recombination conditions.^[^ ^68^ ^]^ However, TEM images taken before and after cyclic annealing (Note S2, Supporting Information) show no significant reduction in TDs within the Ge/Si structures, a finding consistently observed across multiple Ge/Si towers. Consequently, a considerable number of TDs persist, emphasizing the limited effectiveness of cyclic annealing in regions beyond the sidewall areas of the patterned layer.^[^ ^69^ ^]^ This limitation is attributed to constraints in dislocation glide distance^[^ ^70^, ^71^, ^72^, ^73^ ^]^ and the interaction radius between dislocations,^[^ ^72^ ^]^ underscoring the need for a scalable defect‐elimination strategy.

To address this, we generated an interfacial 2D free surface at the Ge/Si structure. Patterned Ge/Si (001) samples, initially exhibiting high TDD, were subjected to bipolar electrochemical etching, as described in refs.[74, 75, 76, 77, 78, 79, 80] The anodic dissolution of Ge and Si proceeds according to the reaction outlined in Note S3 (Supporting Information). The primary challenge with this approach is controlling the electrochemical etching to orient it parallel to the interface, selectively targeting the misfit dislocation network without compromising the integrity of the Ge layer.

**Figure 3:**
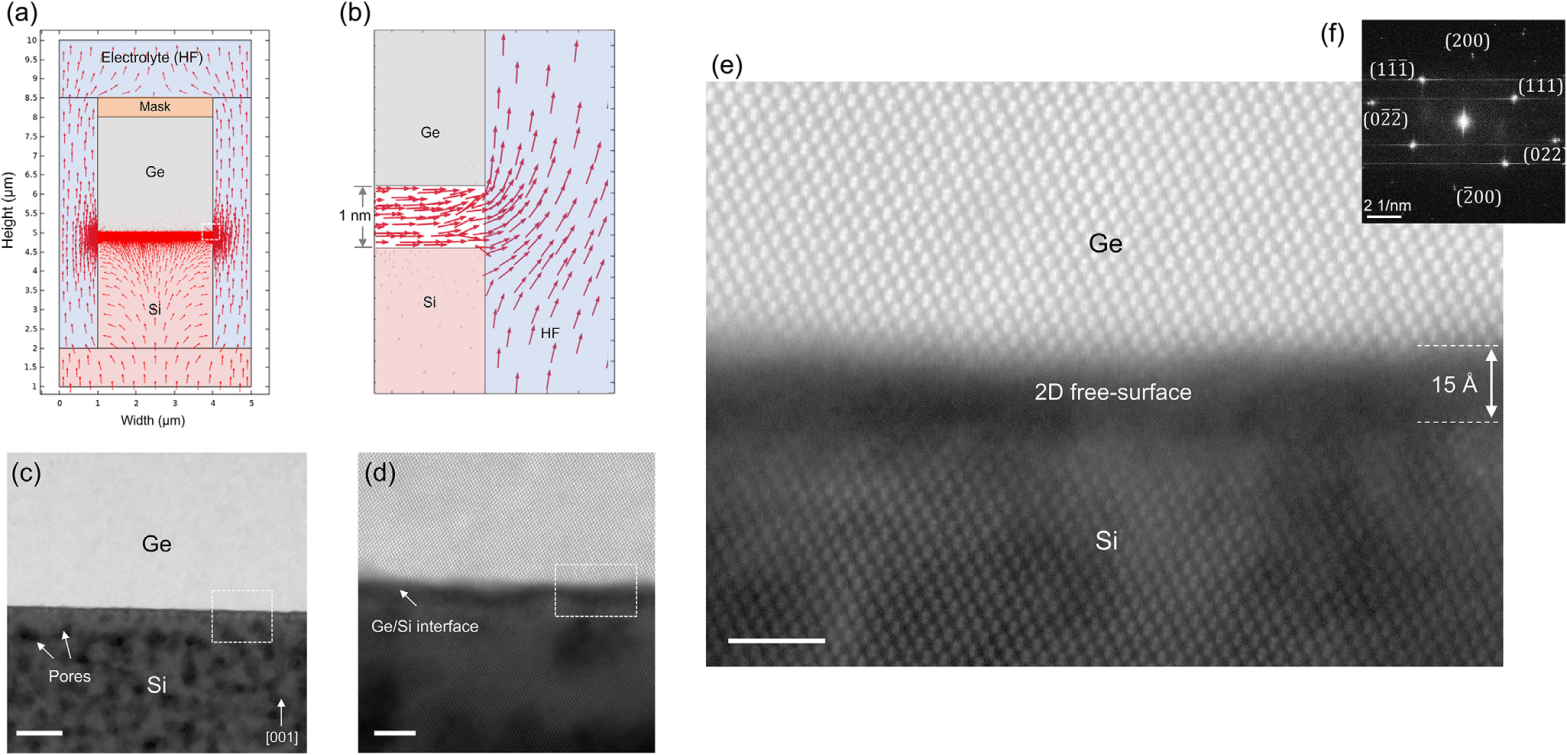
Formation of an interfacial 2D free surface in square‐shaped Ge/Si mesas. a) Finite element simulation of the anodization process, illustrating how tailored doping levels guide current flow during electrochemical etching. The simulation assumes a carrier density of 10^15^ cm^−3^ in Ge and 10^18^ cm^−3^ in p‐type Si, resulting in current primarily confined to the Si. b) (inset of a), Zoomed‐in view of the current streamlines near the Ge/Si boundary, favoring the selective electrochemical etching of misfit dislocations, resulting in the formation of a 2D free surface at the Ge/Si interface. c) HAADF‐STEM image showing pore formation occurring mainly within the Si, with no threading dislocations observed in the Ge layer. d) Magnified view highlighting the continuous 2D free surface formed through preferential etching at the interface, confirming the simulation results. e) Atomic‐resolution image revealing a ≈15 Å nanoscale gap that physically separates the Ge layer from the Si substrate. f) FFT patterns from a selected area of the Ge layer confirms its monocrystalline quality. Scale bars 20 nm (c). Scale bars: 5 nm (d) and 2 nm (e).

To achieve this, we optimized the carrier densities in both the Ge layer and the Si substrate. This engineered doping allowed the electric field lines to penetrate the Si substrate and align horizontally along the Ge/Si interface, facilitating selective etching of the misfit dislocations. **Figure** **3**a presents a finite element simulation of the Ge/Si anodization process, showing how the tailored doping modulates current flow during electrochemical etching. Key parameters, such as etching rate and porosity, were calibrated by adjusting the current density in response to varying Si carrier concentrations, as detailed in Note S4 (Supporting Information). To protect the Ge layer surface from etching, we applied an HF‐resistant mask, while the low carrier concentration in the Ge layer further provided bulk passivation. The electrical model, with carrier densities of 10^15^ cm^−3^ in Ge and 10^18^ cm^−3^ in p‐type Si, demonstrates that the current predominantly flows through the Si substrate. The inset (zoomed in Figure 3b) highlights the concentration of current lines at the Ge/Si interface, driving selective etching of the misfit dislocations and enabling the formation of a 2DFS at the interface. Details of the electrical model are presented in Note S5 (Supporting Information). The preferential electrochemical etching at dislocation sites stems from impurity trapping at the dislocation cores, which locally increases doping concentration and induces a shift in the Fermi level.^[^ ^81^ ^]^ This shift reduces the energy barrier for dislocation core dissolution, accelerating etching near dislocations and promoting the effective removal of the misfit dislocation network.

A complementary mechanism that may drive this localized dissolution is the lattice distortion surrounding the dislocation cores, extending over several atomic distances. The stress field produced by this distortion facilitates the dissolution of lattice elements near the dislocation cores more readily than in unstressed, undistorted regions,^[^ ^82^ ^]^ potentially accounting for the etched interface of ≈15 Å.

Following this, a soft annealing at 500 °C for 1 min was applied to promote the recombination of residual TDs segments without roots (misfit dislocations) and restore the bulk order in the Ge layer. Figure 3c shows High‐Angle Annular Dark‐Field Scanning Transmission Electron Microscopy (HAADF‐STEM) images of the Ge/Si interface, revealing pore formation primarily in the Si substrate due to its high doping concentration, while no TDs are observed in the Ge layer. The inset (zoomed in Figure 3d) shows a continuous 2D free surface region formed through selective etching at the interface, consistent with simulation results. Atomic‐resolution imaging in Figure 3e indicates that the 2D free surface is ≈15 Å in dimension, distinctly separating the Ge layer from the Si substrate. Energy‐dispersive X‐ray (EDX) profiling analysis presented in Note S6 (Supporting Information) shows the relative abundance of oxygen in the nanometric interfacial layer, alongside sharp transitions in the signals for Si and Ge within the 15 Å interfacial region. The presence of oxygen in the EDX profile is due to the increased density of reactive sites (dangling bonds) on both the Si and, especially, on the Ge side due to its high reactivity with oxygen, making them readily available for post‐process bonding. The fast Fourier transform (FFT) patterns from the interface exhibit an amorphous signal in the 2DFS region, likely originating from oxygen atoms. This amorphous interfacial layer is thus more plausibly interpreted as a post‐annealing feature, formed through ambient exposure of these reactive dangling bonds at both Ge and Si surfaces. Importantly, it emerges only after the dislocation dynamics have taken place during annealing, where dislocations are driven toward nearby free surfaces and eliminated through energy minimization.^[^ ^44^ ^]^ While not directly involved in the dislocation recombination process, the presence of this thin oxide may act as a stabilizing barrier, aligning with earlier reports on oxide‐mediated dislocation trapping and blocking in heteroepitaxial systems.^[^ ^83^, ^84^, ^85^ ^]^ In contrast, the FFT patterns from a selected area of the Ge layer, shown in Figure 3f, confirm its monocrystalline quality.

**Figure 4:**
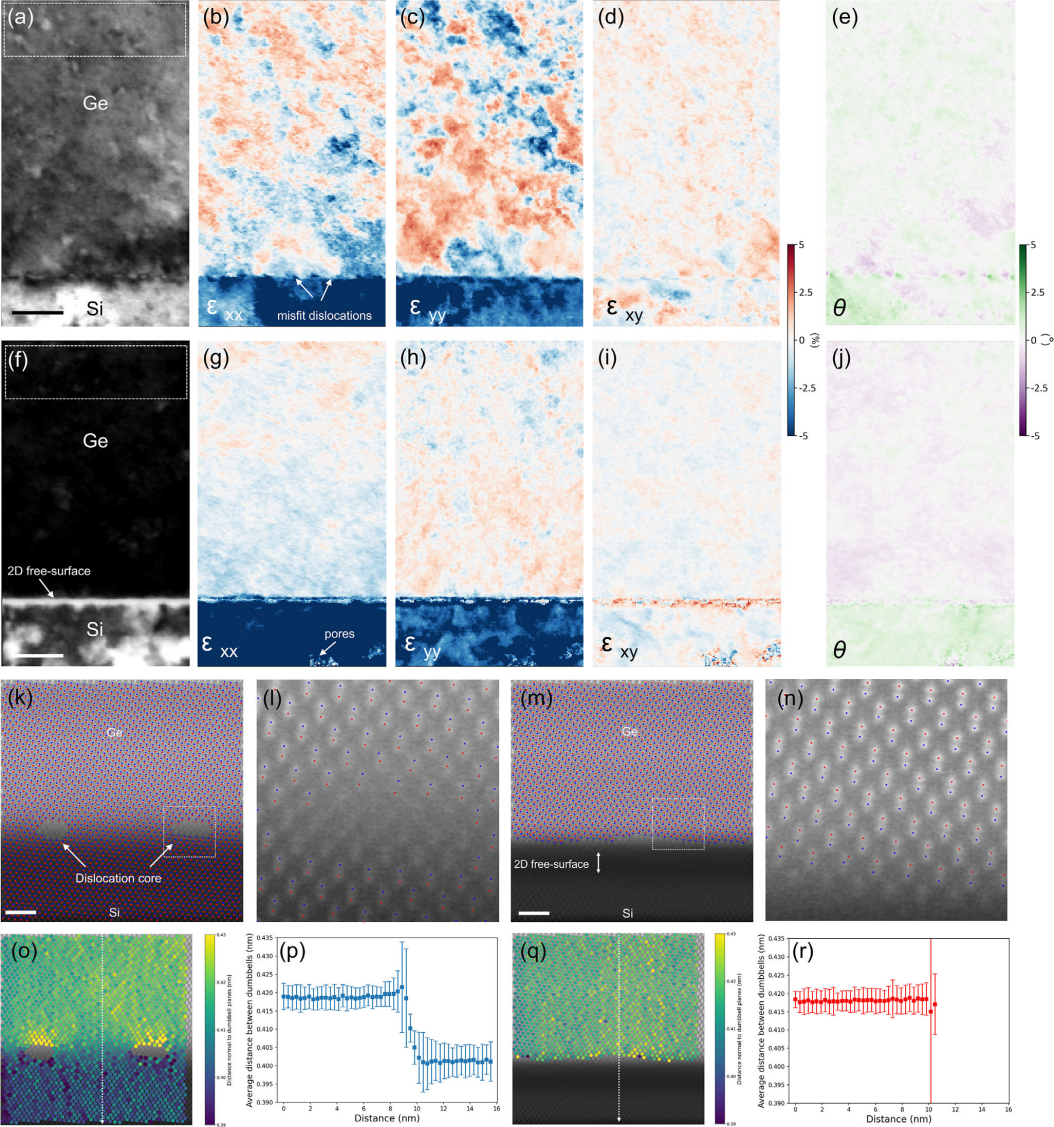
4D‐STEM and atomic‐scale strain mapping of Ge/Si heterostructures with and without a 2D free surface. a) 4D‐STEM virtual bright‐field image of the Ge/Si reference sample, and b–e) its 2D strain components (ε_xx_, ε_yy_, ε_xy_) and 2D rotation maps (θ) at the Ge/Si interface, obtained by quantifying all Bragg peaks in reciprocal space at each scanning position. f) 4D‐STEM virtual bright‐field image of the Ge/Si 2DFS sample, and g–j) its 2D strain components (ε_xx_, ε_yy_, ε_xy_) and 2D rotation maps (θ) at the Ge/Si 2DFS interface, extracted using the same method. Dashed box indicates the reference region used for strain mapping. The color scale bar represents the strain percentage. k) Refined atomic positions overlaid on a high‐resolution STEM image of the Ge/Si reference interface, revealing two misfit dislocation cores (indicated). l) Magnified view of the boxed region in k, showing local lattice distortion at the dislocation core. m) Refined atomic positions at the Ge/Si interface in the 2DFS sample, demonstrating a structurally coherent and defect‐suppressed interface. n) Magnified view of the boxed region in m, highlighting Ge atoms near the 2D free surface that are not bonded to the underlying Si atoms. o) Map of the inter‐dumbbell spacing normal to the dumbbell planes in the Ge/Si reference sample, with clear dilation observed in the vicinity of the dislocations. p) Line profile extracted along the dumbbell planes, averaged through all the dumbbell positions, confirming strain perturbation induced by the dislocations. q) Corresponding inter‐dumbbell spacing map in the 2DFS sample, showing uniform spacing across the interface. r) Line profile extracted from q) indicating consistent strain distribution, with only minor deviations in the outermost atomic layers. The post‐processing were carried out using the 4D‐STEM package. Scale bars 20 nm (a,f) and 2 nm (k,m).

**Figure 5:**
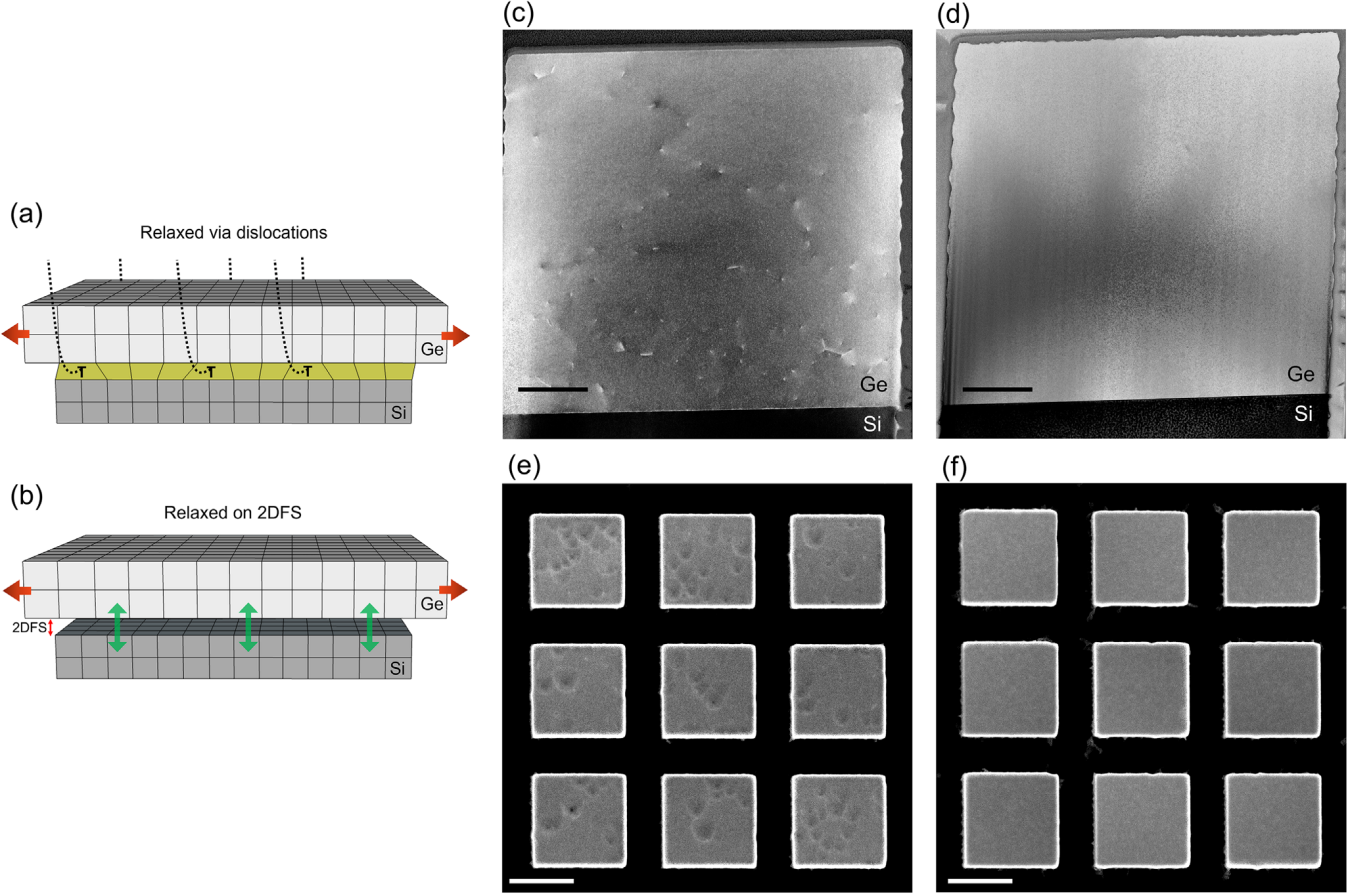
Assessment of defect density. a) Schematic representation of strain relaxation through dislocations, where 'T' marks the formation of a misfit dislocation. b) Schematic illustration of strain relaxation on a 2D free‐surface structure (2DFS). c) Dark Field TEM images of the square‐shaped Ge/Si from reference sample and d) from the 2DFS sample, with the latter showing the absence of threading dislocations. e) Etch‐pit densities at the top of the square‐shaped Ge/Si reference sample, indicating a high density of threading dislocations, and f) from the 2DFS sample, which is free of pits. These observations confirm the effective annihilation of dislocations by introducing the 2D free surface at the Ge/Si interface. Scale bars: 500 nm (c,d); 2 µm (e,f).

To understand the TDs removal mechanism in this novel structure, strain mapping at the interface was conducted by quantifying all the Bragg peaks in diffraction space at each location using 4D‐STEM. **Figure** **4** presents the results for both the Ge/Si reference and the Ge/Si 2DFS samples. Strain field components ε_xx_, ε_yy_, and ε_xy_, together with the corresponding virtual bright‐field images (Figure 4a,f), are shown in Figure 4b–d,g–i, respectively, with rotation maps θ in Figure 4e,j. The strain maps for the Ge/Si 2DFs sample reveal a globally uniform deformation field across the Ge regions (Figure 4b,c). Given the 4.2% lattice mismatch between Ge and Si, significant strain relaxation is expected in the Ge layer due to the presence of the 2DFS region. In the reference sample, localized strain concentration was observed in the Ge layer (Figure 4g,h). Note that dislocation cores are visualized in the strain maps as blue regions at the interface (also refer to the geometric phase analysis results in a zoomed‐in view in Figure S6 (Supporting Information), where the core structures of misfit dislocations are distinctly observed through the strain lobe pairs^[^ ^86^ ^]^). The strain distribution within the Ge layer is notably non‐uniform across all components due to these TDs, with relaxation predominantly occurring through dislocation formation.^[^ ^87^ ^]^ In contrast, the Ge/Si 2DFS sample, after eliminating the misfit dislocation network and undergoing a soft annealing process, shows relaxed Ge crystal, effectively decoupled from the strain imposed by the Si substrate. The strain maps confirm the absence of TDs, as evidenced by the uniform strain distribution throughout the Ge layer. Features observed in the Si substrate are attributed to pores formed during electrochemical etching. The strain profiles in the x and y directions at the high‐magnification interface were analyzed in both samples (Note S7, Supporting Information). Strain analysis indicates that the Ge layer in both the Ge/Si reference and 2DFS samples exhibits comparable ε_xx_ and ε_yy_ strain levels relative to their respective substrates. However, unlike in the bulk Si, the strain profiles undergo an abrupt drop across the 2DSF, suggesting a higher degree of relaxation compared to Ge grown directly on bulk Si. To further investigate the local strain behavior and structural coherence at the atomic scale, we performed high‐resolution atomically resolved strain mapping by refining the atom positions across the Ge/Si interface and quantifying the interatomic distances (Figure 4k–q). In the Ge/Si reference sample, the atomic‐resolution STEM image reveals two well‐defined misfit dislocation cores at the interface (Figure 4k), with significant lattice distortion localized in their vicinity (Figure 4l). Quantitative maps of the inter‐dumbbell spacing normal to the dumbbell planes (Figure 4o) and the corresponding line profile (Figure 4p) confirm a clear dilation of the Ge lattice at these dislocation sites, indicating local strain perturbation induced by the misfit dislocations. In contrast, the 2DFS sample exhibits a structurally coherent interface, free of visible dislocations, with refined atomic columns showing a well‐ordered lattice (Figure 4m). A zoomed‐in view of the 2D free surface region (Figure 4n) shows Ge atoms that are not bonded to the underlying Si, suggesting effective interface decoupling. The corresponding inter‐dumbbell spacing map (Figure 4q) and line profile (Figure 4r) reveal a uniform strain distribution, with only minor deviations near the outermost atomic layers. These findings provide direct evidence that the 2D free surface architecture suppresses dislocation formation and facilitates strain relaxation via a non‐dislocation‐mediated pathway—notably through interfacial bond rupture rather than dislocation release, as further illustrated in **Figure** **5**a,b.

To assess the TDD, cross‐sectional TEM images were analyzed in various regions across multiple regions of the sample and several Ge/Si towers. Each lamella traverses more than eight Ge/Si towers, with each tower thinned to a different extent to reveal dislocation segments (Note S8, Supporting Information). Figure 5c, d presents TEM images of full Ge/Si towers from reference and 2DFS samples, respectively. The contrast in the images is primarily influenced by thickness fringes and bend contours, typical of crystalline cross‐sectional samples. Dark points and segments indicate strain‐induced bending caused by dislocations. The Ge/Si reference sample exhibits a notably high dislocation density, while no TDs are observed in the 2DFS sample area, as seen in Figure 5d. Despite the clear distinction between the two samples, the absence of dislocations in the TEM images of the 2DFS sample does not provide a fully quantitative measurement—due to the potential underestimation of bulk dislocations resulting from TEM sample thinning. Additionally, fabricating multiple TEM samples with different orientations to fully quantify dislocations in a Ge/Si tower is more complex. Nevertheless, this technique has provided an overall view of the quality of the probed Ge/Si towers. To achieve a more precise quantification of TDD, EPD analysis was performed across multiple Ge/Si towers. Both Ge/Si and 2DFS samples were immersed in a solution composed of two parts 49 wt.% HF and one part 0.1 M K_2_Cr_2_O_7_, which selectively etched mixed and screw dislocations in the Ge layer. SEM images of the EPD for both samples are shown in Figure 5e,f. Pit counts revealed a high TDD of ≈10^18^ cm^−2^ at the surface of the Ge/Si reference sample. In contrast, the 2DFS sample's surface appeared entirely pit‐free, indicating no TD dislocations reaching the surface across all towers.

While no TDs were observed across the majority of the 2DFS sample, within the statistical limits of our survey, complete elimination of lattice defects cannot be definitively claimed. Nevertheless, multi‐scale analyses, including plan‐view TEM and EPD (Note S9, Supporting Information), consistently revealed no observable dislocations across multiple locations. This robust absence of extended defects underscores a significant enhancement in the crystallinity of the Ge layer, validating the effectiveness of the 2DFS strategy in suppressing defects in mismatched heteroepitaxial systems.

Building on the results obtained from GPA, which demonstrate the complete relaxation of the Ge layer and confirm its high crystalline quality through assessments of dislocation‐free characteristics via TEM and EPD analysis—despite the significant lattice and thermal mismatch with the Si substrate—it is important to emphasize the defect elimination mechanism promoted by the 2DFS. We propose that the removal of dislocation roots from the interface and the formation of a Ge‐on‐quasi‐nothing structure leave little opportunity for TDs to exist within the Ge layer, particularly following the corrective annealing of microcrystals. During this annealing, multiple mechanisms may contribute to the elimination of rootless TDs. One prominent mechanism is the role of the 2DFS formed at the Ge/Si interface, which acts as a free surface, inhibiting dislocation propagation. TDs near the interface bend downward and glide toward the 2DFS to minimize their length.^[^ ^44^, ^88^ ^]^ Those dislocations within the Ge layer are annihilated at the 2DFS rather than propagating toward the surface. Elimination in pairs is another plausible mechanism. Under a thermal driving force, dislocations can interact with others or defects within their field. In the Ge/Si (001) system, dislocation arms lie on the (111) and (11−1) slip systems. TD termination occurs when two or more dislocations with opposite Burgers vectors and equal magnitude reach the void, resulting in a net Burgers vector of zero, as per Frank's rule of conservation.^[^ ^68^ ^]^ Several reactions can occur in cubic semiconductors, such as fusions and partial or complete annihilation. For instance, TD segments on the same slip plane (e.g., a/2[1–10] and a/2[−110]) or on parallel planes (e.g., a/2[01−1] and a/2[0−11]) can annihilate, while TD fusion (e.g., a/2[10–1] and a/2[011]) results in a single TD with a/2[110].^[^ ^89^ ^]^ Another mechanism involves singular TD segments which can glide^[^ ^90^ ^]^ or climb^[^ ^91^, ^92^ ^]^ within the Ge microcrystals and annihilate at the four surrounding free surfaces, yielding a defect‐free Ge crystal.^[^ ^93^ ^]^ Another noteworthy aspect is that the stability of the seemingly free‐standing Ge layer on a 2D‐free surface is maintained through precisely controlled electrochemical etching conditions, resulting in a silicon porosity of less than 60% (Figure S3, Supporting Information). With a 2D free surface underlayer of ≈15 Å, the electrostatic potential of the substrate remains significant, interacting indirectly with the atoms of the epilayer. While no direct chemical bonds form, this interaction ensures the retention of the Ge layer, without necessitating adherence to the substrate's crystallographic orientation, as shown in Figure 5b. A similar phenomenon is observed in remote epitaxy, where epitaxial growth is influenced by substrate bonding ionicity or polarity through transparent 2D interlayer.^[^ ^31^, ^94^ ^]^ Another possibility is that the Ge layer may be stabilized through nanoscale connections between Ge and Si via covalent bonds, which, although partial, do not generate sufficient strain to induce dislocation formation. This mechanism is similar to pinhole‐based epitaxy, where nucleation occurs through pinholes in a 2D layer, followed by lateral overgrowth.^[^ ^27^ ^]^ However, an analysis of multiple TEM images of the Ge/Si interface revealed no evidence of a direct connection between the Ge and Si layers.

**Figure 6:**
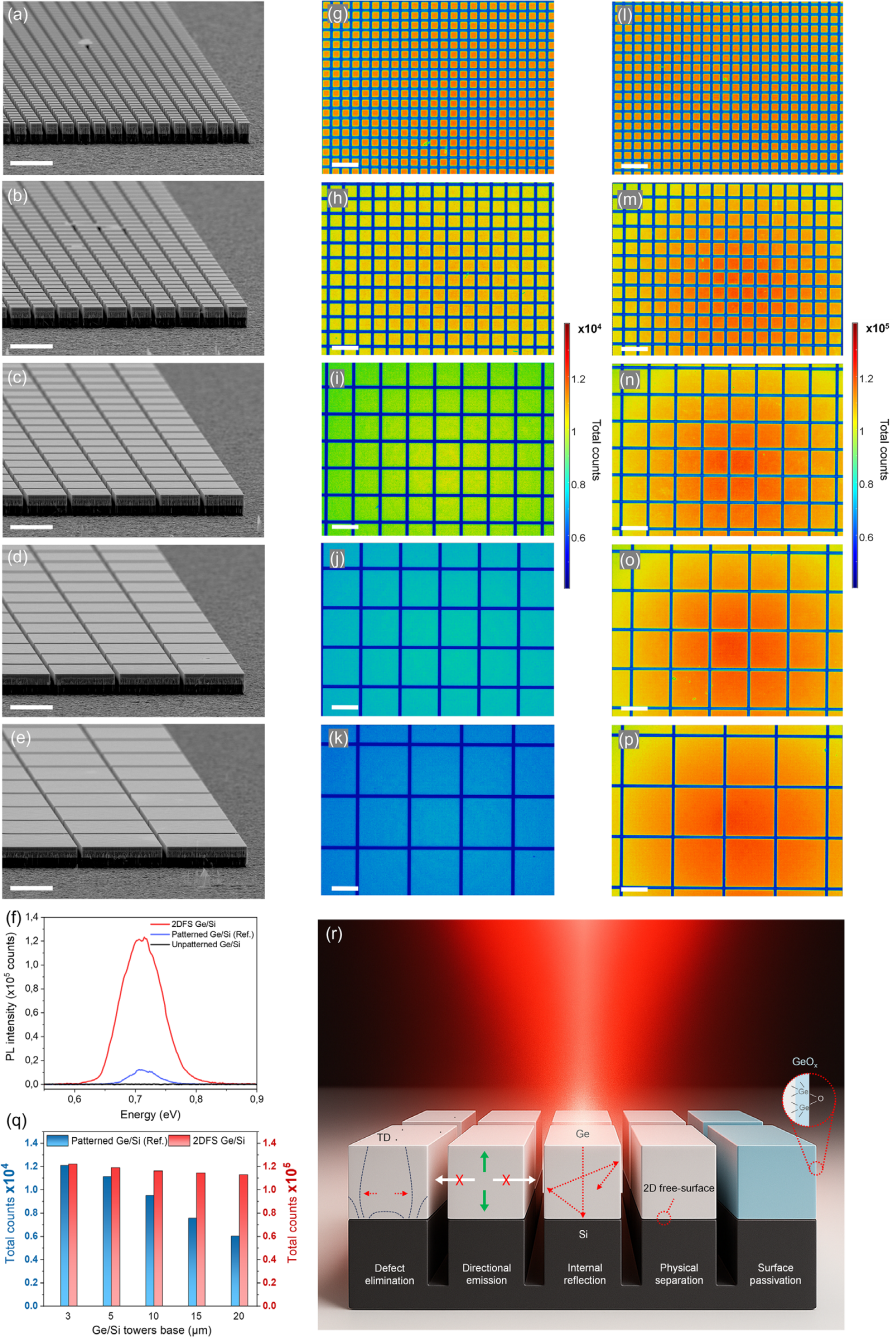
Scaling and optical properties through Hyperspectral Photoluminescence (PL) imaging. a–e) SEM images of matrices of square‐shaped Ge/Si samples with increasing sizes: 3 × 3, 5 × 5, 10 × 10, 15 × 15, and 20 × 20 µm^2^, respectively. f) Room temperature PL spectra of 3 × 3 µm^2^ square‐shaped Ge/Si reference samples, 2DFS samples, and unpatterned Ge/Si samples. i–k) Integrated hyperspectral PL images of square‐shaped Ge/Si reference samples, and l–p) 2DFS samples. q) Steady‐state PL intensity from a central region of maps of Ge/Si reference samples, as a function of tower base dimensions, compared with 2DFS samples. (r) Schematic illustration of the synergistic physical mechanisms contributing to the enhanced PL in 2DFS‐enabled Ge/Si micropillars. Scale bars 10 µm (a–p).

These findings collectively affirm the effective accommodation of strains and the successful elimination of TDs on a 2D free surface. This configuration, independent of substrate constraints, enables the growth of defect‐free Ge microcrystals measuring 3 × 3 µm^2^, motivating us to scale this technique for larger areas. Accordingly, we fabricated arrays of square‐shaped Ge/Si samples with dimensions of 3 × 3, 5 × 5, 10 × 10, 15 × 15, and 20 × 20 µm^2^, as illustrated in **Figure** **6**a–e. Subsequently, we examined the optical properties of the epitaxial Ge layer both with and without the 2DFS. Figure 6f presents the steady‐state room‐temperature PL spectra for 3 × 3 µm^2^ square‐shaped Ge/Si tower arrays, of both reference and 2DFS samples. For comparative analysis, the spectrum obtained from the unpatterned region of the same sample is represented by the black line. Continuous Ge/Si layers exhibit notably low external quantum efficiency (EQE) due to two primary factors. First, a significant portion of the light generated within the Ge epilayer is trapped by total internal reflection, which severely limits light extraction efficiency.^[^ ^95^ ^]^ Second, the high density of defects in the continuous Ge layer above unpatterned substrate areas adversely affects internal quantum efficiency, as the carrier dynamics are dominated by parasitic nonradiative channels. This combination of factors leads to a luminescence intensity that falls below the measurement noise threshold.^[^ ^96^ ^]^ In contrast, Figure 6f demonstrates a pronounced increase in PL intensity for the Ge/Si towers reference samples, consistent with emissions recorded from Ge microcrystals grown epitaxially on Si pillars.^[^ ^95^ ^]^ Remarkably, the EQE of the 2DFS configuration exhibits a substantial increase, surpassing that of the patterned Ge/Si reference by an order of magnitude. The interband emission intensity in the Ge/Si 2DFS is found to be comparable to the PL intensity of ultra‐low dislocation Ge wafers.^[^ ^97^ ^]^ The broad PL emission observed in the 2DFS sample likely results from a convolution of band‐edge and interface‐related recombination pathways. While dislocation‐related PL near 0.67 eV^[^ ^98^, ^99^, ^100^ ^]^ cannot be entirely ruled out, the absence of detectable TDs in TEM and EPD analyses suggests it is not the dominant contribution. At room temperature, thermal broadening and residual strain may cause low‐temperature features to merge, such as direct‐gap and phonon‐assisted transitions.^[^ ^97^, ^101^ ^]^ Temperature‐dependent PL measurements would help clarify the contribution of each recombination channel.^[^ ^102^ ^]^ Figure 6g–k,l–p compare the spatial PL intensities of Ge/Si reference and 2DFS arrays, respectively, as measured by hyperspectral microscopy. Figure 6q displays the PL counts from the central regions of the maps for both Ge/Si reference and 2DFS arrays as a function of tower base dimensions. The non‐homogeneous distribution observed in certain maps is attributed to Bragg scattering effects,^[^ ^103^ ^]^ as previously noted by cathodoluminescence.^[^ ^104^ ^]^ The PL intensity of the Ge/Si reference towers shows a pronounced decline with increasing tower size, which can be attributed to the two factors mentioned earlier regarding PL emission from continuous or large‐dimension Ge/Si layers. In contrast, Ge/Si 2DFS arrays exhibit a tenfold increase in PL intensity compared to the Ge/Si reference, maintaining remarkable optical stability even as tower dimensions are scaled up. This substantial enhancement stems from the convergence of several synergistic mechanisms: i) strain relaxation and defect suppression through 2D free‐surface engineering, which mitigates nonradiative recombination and enhances internal quantum efficiency;^[^ ^95^ ^]^ ii) modification of the local photonic environment induced by the semi‐floating geometry, promoting directional emission and improving light extraction;^[^ ^102^ ^]^ iii) optical confinement effects associated with the 3D architecture, enabling multiple internal reflections and extended optical path lengths, thereby increasing both absorption and radiative recombination rates;^[^ ^105^ ^]^ iv) suppression of carrier extraction following physical separation from the heavily doped Si substrate, which reduces parasitic carrier loss;^[^ ^106^ ^]^ and v) surface passivation via native GeO_x_ formation, which lowers surface recombination velocity.^[^ ^107^ ^]^ These interacting effects, schematically illustrated in Figure 6r, account for the pronounced PL enhancement. This stability arises from the 2DFS architecture's ability to eliminate defects and engineer the local photonic environment of the Ge layer—via strain relaxation, carrier confinement, and enhanced light extraction—resulting in a substantial boost in emission efficiency.

## 3 Conclusion

We report our discovery of an alternative pathway for relaxing misfit strain in heteroepitaxial films, achieved through the selective severing of atomic bonds between the epitaxial layer and substrate. This innovative approach results in the formation of a 2D free‐surface interfacial region ≈15 Å thick. Our experimental findings indicate that the removal of atomic rows at the interface, which host misfit dislocations, endows the epilayer with a degree of freedom that enables spontaneous self‐healing under thermal driving forces. This approach has been validated in a highly mismatched system—specifically, Ge on Si with a 4.2% lattice misfit—where we observe a substantial reduction in dislocation density. Additionally, we have investigated potential mechanisms that contribute to the elimination of TDs. Furthermore, hyperspectral PL measurements reveal a significant enhancement in emission efficiency in Ge/Si towers with scalable dimensions, thereby confirming the effective suppression of TDs. The scalability of this technique has been evaluated at the wafer level, demonstrating high uniformity across a 3‐inch substrate (Note S10, Supporting Information). Additionally, spreading resistance profiling confirms that the electrochemical etching process preserves the carrier concentration within the Ge layer, indicating that the structural decoupling achieved via 2DFS does not compromise electronic integrity (Note S11, Supporting Information). Complementary thermal cycling experiments further demonstrate the mechanical robustness of the 2DFS‐enabled heterostructures, with no observable degradation over multiple heating and cooling cycles (Note S12, Supporting Information). While demonstrated here for Ge/Si, the 2DFS strategy may, in principle, be extended to other heteroepitaxial systems, including group‐IV alloys such as GeSn/Si and SiGe/Si, and possibly to III–V/Si integration using this approach as a structurally and chemically compatible buffer. The 2DFS interface offers a promising route for accommodating lattice mismatch without generating dislocations, provided that etching selectivity and carrier concentration asymmetry are maintained across the interface.

Although further research is warranted to elucidate the mechanisms underlying dislocation elimination, this relaxation strategy represents a considerable advancement toward the monolithic integration of materials characterized by large lattice mismatches and minimized dislocation densities. This pivotal discovery has the potential to broaden the material palette for next‐generation electronics and photonics applications.

## 4 Experimental Section

### Fabrication of Ge/Si Tower Arrays

Ge/Si tower arrays were fabricated using a combination of electron beam (e‐beam) lithography and reactive ion etching on commercial Ge/Si wafers oriented along the <100> direction with Ge thickness of 3 µm. These wafers were p‐type boron‐doped, with a carrier density of 10^15^ cm^−3^ in Ge and 10^18^ cm^−3^ in the Si substrate, exhibiting a TDD of ≈10^8^ cm^−2^. The Ge/Si arrays were fabricated with varying diameters of 3 × 3, 5 × 5, 10 × 10, 15 × 15, and 20 × 20 µm^2^. E‐beam lithography was conducted at an acceleration voltage of 100 keV, utilizing a specially adapted e‐beam writer that implemented resolution modulation—employing lower resolution in the center of the pattern and higher resolution at the edges to achieve well‐defined features. A 500 nm thick positive resist was employed, which was sensitive to e‐beam exposure and resistant to hydrofluoric acid for subsequent steps. After exposure, the resist was developed in methyl isobutyl ketone (MIBK) for 2 min. The etching process for Ge/Si was performed using an inductively coupled plasma method within an Advanced Etching DRIE system. This process utilized a mixture of fluorinated gases, with sulfur hexafluoride (SF_6_) for Ge/Si etching, octafluorocyclobutane (C_4_F_8_) for sidewall passivation, and oxygen (O_2_) for process enhancement. The etching of the Ge/Si towers was executed through a dual‐step method of simultaneous etching and passivation, where the gases for etching and passivation were alternated in brief cycles to achieve deep etching of the Ge/Si structures, following the Bosch process. Following the Bosch etching, an additional plasma etching step employing only SF_6_ was implemented to eliminate residual silicon micro‐masking. Further surface cleaning was conducted to remove any deposits left from the plasma etching process, ensuring the integrity of the fabricated structures.

### Selective Electrochemical Etching

Selective electrochemical etching was performed using a bipolar electrochemical etching process within a two‐electrode cell, employing a platinum wire as the counter electrode. The electrolyte consisted of a 5:1 mixture of (HF, 49%) and anhydrous ethanol. For the selective etching of the Ge/Si samples, alternating anodic and cathodic currents were applied with a density of ±50 mA cm^−^^2^, utilizing pulse durations of 1 s over a total duration of 1 min for Ge/Si towers measuring 3 × 3 µm^2^. Throughout the etching process, the surfaces of the Ge/Si tower were protected by the resist mask. After etching, this mask was removed by immersing the samples in acetone for one night.

### Thermal Annealing

The thermal annealing of Ge/Si towers was conducted in a forming gas atmosphere (N_2_‐H_2_ 90:10) using a J.I.P. ELEC JetFirst rapid thermal annealing system, operating at a ramp rate of 10 °C s^−1^. Two annealing recipes were employed: a cyclic annealing process was applied to evaluate its effectiveness in reducing the dislocation density in the Ge/Si towers in reference sample under the same process condition used in refs. [66, 67] For 2DFS process, a soft anneal at 500 °C for 1 min was utilized to eliminate any residual threading dislocations after the selective etching of the interface.

### Structural Characterization by TEM

Structural characterization was conducted using a scanning/transmission electron microscope operating in high‐angle annular dark field mode. The analysis was performed on a Titan Themis microscope, functioning at an accelerating voltage of 200 kV, equipped with a CEOS probe corrector and a Ceta 16M camera from FEI. Prior to S/TEM examination, samples underwent focused ion beam (FIB) thinning and ion milling for preparation. To protect the surface during the FIB process, a 100 nm‐thick carbon layer was deposited. Elemental distribution analysis was performed using Gatan digital micrograph (DM) software alongside EDX integrated with STEM.

### GPA Calculation

The strain analysis was performed using TemCompanion and custom Python scripts. The theoretical framework and methods follow the approach described in ref. [86] For the GPA, two strong {111} diffraction spots were selected, with an aperture radius of ≈3 nm (real‐space equivalent). To minimize edge effects, a cosine function was applied to create a smoothed aperture edge, with a smoothing factor of 0.3 (i.e., the function begins damping from 70% to 100% of the aperture radius). The strain tensors were computed in image coordinates, where ε_xx_ corresponds to the horizontal direction and ε_yy_ to the vertical direction. The entire image was used to define the reference g vectors, setting the average lattice spacing of Si and Ge to approximately zero strain.

### STEM and Atomic‐Scale Strain Analysis

4D‐STEM and atomic‐resolution STEM imaging were performed using a Thermo Fisher Scientific Spectra 300 probe‐corrected S/TEM operated at 300 kV. 4D‐STEM dataset were acquired using an electron microscope pixel array detector (EMPAD) with a beam convergence angle of 1.5 mrad. Post‐processing and strain analysis were carried out using the py4DSTEM package [108]. High‐angle annular dark‐field (HAADF) images were acquired using a beam convergence angle of 30 mrad with a collection range of 63–200 mrad. To minimize drift and scan jitter, images were recorded as a scan series of 20 frames (1024 × 1024 pixels, 200 ns pixel time), which were subsequently aligned and averaged using the *TemCompanion* software.^[^ ^109^ ^]^ Atom position refinement was performed using the *Atomap* package,^[^ ^110^ ^]^ followed by interatomic distance analysis with custom Python scripts. Prior to atom detection and refinement, all images were filtered to improve the signal‐to‐noise ratio using the average background subtraction algorithm from the *hrtem_filter* package.^[^ ^111^ ^]^

### Etch‐Pit‐Density Measurement

For etching purposes, the etchant consisted of a mixture of two volumetric parts of 49 wt.% HF and one part of 0.1 M K_2_Cr_2_O_7_. Etch pits were quantified on the top surface by analyzing SEM images, and the results were averaged over multiple samples.

### Hyperspectral PL Measurement

PL measurements were performed at room temperature using a hyperspectral imaging system equipped with a lead sulfide (PbS) detector. The excitation source was a continuous‐wave Nd‐YVO4 laser operating at 1.165 eV. The laser spot size on the sample surface was ≈150 µm in diameter, with a power density between 0.5 and 2 kW cm^−^^2^. The laser was set up to provide uniform illumination across the entire field of view, allowing for global imaging. PL signals were captured through a 20× objective and directed onto a CCD camera via a volume Bragg grating, with a custom script enhancing the resolution of the PL maps.

## Conflict of Interest

The authors declare no conflict of interest.

## Supporting information



Supporting Information

## Data Availability

The data that support the findings of this study are available in the supplementary material of this article.
