# Supplementary material for: Overcoming Material Incompatibility via 2D Free‐Surface Engineering
Source: Adv Mater. 2025 Aug 11;37(47):e05101. doi: 10.1002/adma.202505101 (PMC12651136; doi:10.1002/adma.202505101)
Supplement: Supplementary file 1 — Supporting Information [file ADMA-37-e05101-s001.pdf]

ADVANCED MATERIALS

Supporting Information

for *Adv. Mater.*, DOI 10.1002/adma.202505101

Overcoming Material Incompatibility via 2D Free-Surface Engineering

Youcef A. Bioud, Meriem Bouchilaoun, Waldemar Schreiber, Redouane Amrar, Gilles Patriarche, Tao Ma, Jens Ohlmann, Ali Soltani, David Lackner and Stefan Janz*

SUPPLEMENTARY INFORMATION

Overcoming Material Incompatibility via 2D Free-Surface Engineering

Youcef A. Bioud [1,2*], Meriem Bouchilaoun [2], Waldemar Schreiber [1], Redouane Amrar [3], Gilles Patriarche [4], Tao Ma [5], Jens Ohlmann [1], Ali Soltani [3], David Lackner [1], Stefan Janz [1]

[1] Division Photovoltaics, Fraunhofer Institute for Solar Energy Systems (ISE), Heidenhofstraße 2, 79110 Freiburg, Germany.
[2] Department of Electrical Engineering, Université de Sherbrooke, Sherbrooke, Québec J1K 2R1, Canada.
[3] Laboratoire Nanotechnologies Nanosystèmes (LN2) - CNRS UMI-3463, Institut Interdisciplinaire d'Innovation Technologique (3IT), Université de Sherbrooke, 3000 Boulevard Université, Sherbrooke, J1K OA5, QC, Canada.
[4] Université Paris-Saclay, CNRS, Centre de Nanosciences et de Nanotechnologies, 91120 Palaiseau, Paris, France.
[5] Michigan Center for Materials Characterization, University of Michigan, Ann Arbor, Michigan 48109, United States.

[*]*Email: y.bioud@USherbrooke.ca*

Supplementary Note. 1:

Plasma etching rate calculation

To demonstrate the dependence of the etch rate on the aspect ratio, we computed the average instantaneous etch rate (ER) as a function of the average instantaneous aspect ratio (AR) using the following equation:

$$ER(AR_i) = \frac{d_i - d_{i-1}}{t_i - t_{i-1}}$$

$$AR_i = \frac{d_i + t_{HM_i} + d_{i-1} + t_{HM_{i-1}}}{2DC}$$

Here, d_i and t_i represent the etched depth and time for the ith experiment, while t_{HMi} denotes the remaining hard mask thickness for the ith experiment. DC refers to the critical dimension, which corresponds to the trench width [1].

Supplementary Note. 2:

Effect of Post-Epitaxial Cyclic Annealing on the Evolution of Dislocation Density

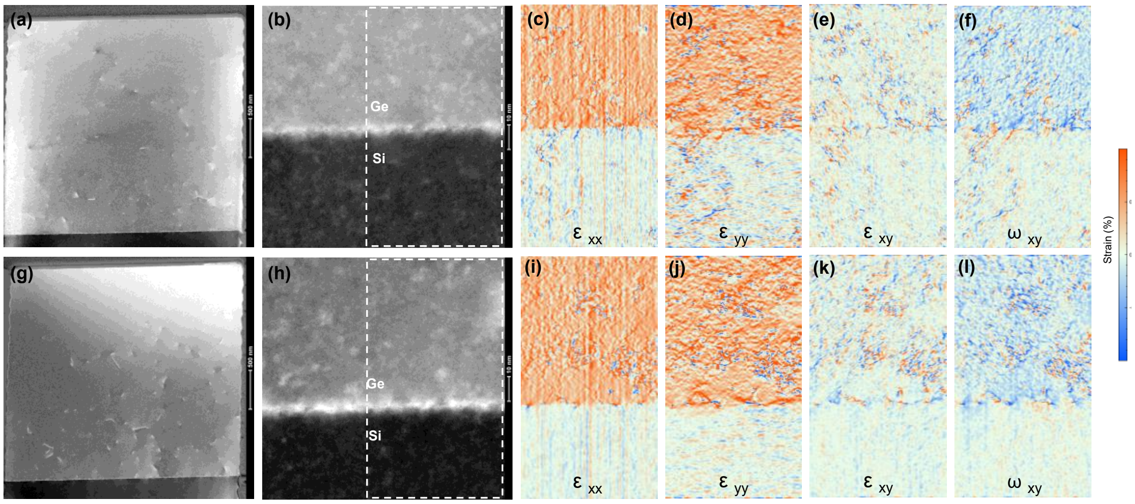

Fig. 1. STEM Dark Field images and GPA analysis of the Ge/Si tower are presented for both the reference sample (a-f) and the cyclically annealed sample (g-l). Panels (a) and (g) reveal the complete tower structures, with threading dislocation segments visible and showing minimal changes between the reference and cyclically annealed samples. The interfaces in panels (b) and (h) exhibit similar characteristics, indicating that the cyclic annealing process has had little impact on the structure. Panels (c-f) display the 2D strain components (ε_{xx}, ε_{yy}, ε_{xy}) and 2D rotation maps (ω_{xy}) at the Ge/Si interface of the reference sample, as determined GPA. Panels (i-l) show the corresponding strain and rotation maps for the cyclically annealed sample, which reveal a high degree of similarity. The strain distribution in the Ge layer is non-uniform across all components, influenced by the threading dislocations (TDs). The 2D strain components are extracted from the region indicated by the dashed lines.

Supplementary Note. 3:
Electrochemical etching reactions

The electrochemical dissolution of Ge in hydrofluoric acid (HF) can proceed via two anodic pathways, determined by the valence state of the dissolving species, which in turn depends on the applied potential [2]. At certain potentials, Ge undergoes divalent anodic dissolution, while at others, tetravalent anodic dissolution occurs. The divalent reaction follows:

$$Ge + 6HF + h^+ \rightarrow GeF_6^{2-} + 4H^+ + H_2 + e^-$$

and the tetravalent dissolution proceeds as:

$$Ge + 6HF + 4h^+ \rightarrow GeF_6^{2-} + 6H^+$$

For the electrochemical dissolution of Si in HF, the associated electrochemical reactions can be described as follows [3]:

$$Si + 2HF + nh^+ \rightarrow SiF_2 + 2H^+ + (2-n)e^-$$

$$SiF_2 + 2HF \rightarrow SiF_4 + H_2$$

$$SiF_4 + 2HF \rightarrow H_2SiF_6$$

Where h^+ is the hole, e^- is the electron, and n is the number of charges exchanged.

Supplementary Note. 4:

Porosity Measurement Using Infrared Reflectance:

When infrared light is directed onto the surface of freshly fabricated porous Si samples, it reflects from both the top interface (air to porous Si) and the bottom interface (porous Si to Si substrate). These reflected waves interfere, producing Fabry-Perot fringes in the reflectance spectrum, as illustrated in Fig. 2. The resulting interference pattern allows for the calculation of the film's refractive index, following the provided equation:

$$n_{PSi} = \frac{m}{2\delta \text{v} t}$$

Where m represents the number of fringes within the wavenumber range, δv, and t denotes the porous layer thickness, which is estimated through SEM images.

Assuming the medium is isotropic, the porosity of the porous Si layer can be effectively determined using the Landau-Lifshitz-Looyenga model [4]. For freshly prepared porous Si, the refractive index is expressed as a weighted average of the refractive indices of Si and air, with the model providing a reliable method to estimate the porosity based on the measured refractive index values. This allows a correlation between the porosity and the optical properties of the material.

$$P = \frac{n_{PSi}^{2/3} - n_{Si}^{2/3}}{1 - n_{Si}^{2/3}}$$

where we have taken the refractive index of air to be unity. The refractive index of Si is reasonably stable around nSi ≈ 3.42 in the range of interest.

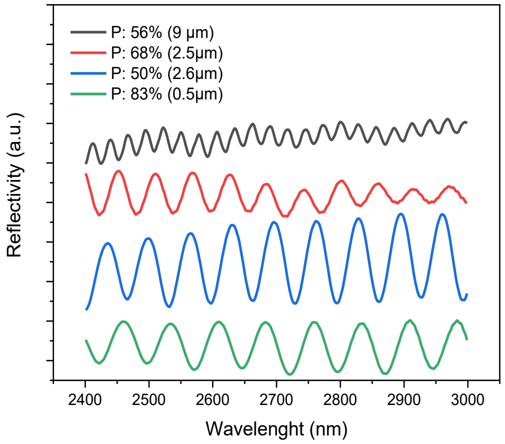

Fig. 2. Reflectivity spectra of randomly porous Si layers fabricated using the BEE technique, exhibiting varying porosities and thicknesses.

Porosity Measurement Using Gravimetric Method

The porosity was further validated through weight measurements conducted before and after the etching process, employing the gravimetric method [5]. The wafers were weighed prior to etching (m_1) and then again after etching (m_2) to quantify the amount of Si removed during the formation of the porous layer. Subsequently, the wafers were immersed in a 1.0 M KOH solution for 10 seconds to selectively remove the porous layer. The final mass of the substrate was recorded after this treatment (m_3). The porosity is then calculated using the following formula:

$$P(\%) = \frac{m_1 - m_2}{m_1 - m_3}$$

Porosity and Etch Rate as a Function of Current Density and Si Carrier Density

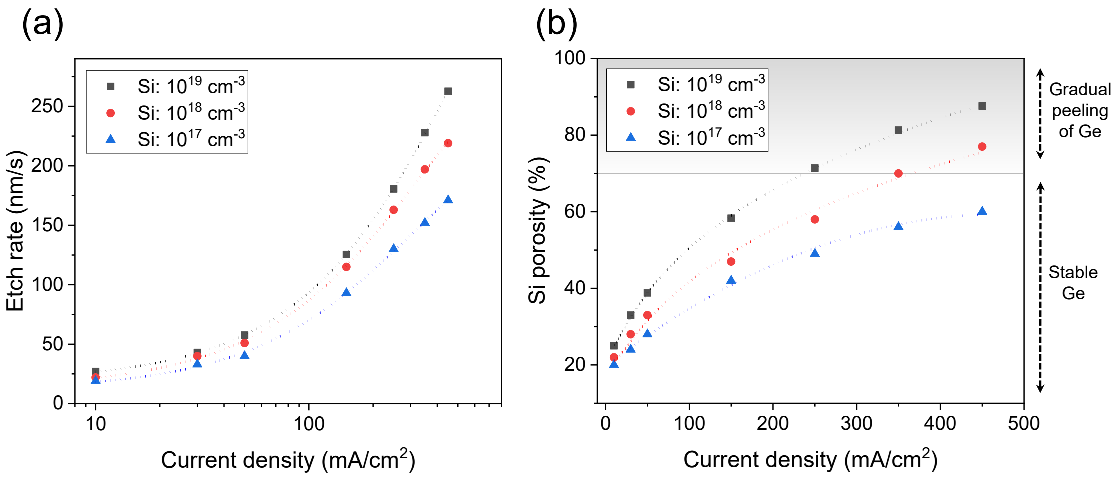

Fig. 3. Si etching rate (a) and Si porosity evolution (b) as a function of current density for various Si carrier densities. The dashed line in (b) indicates the porosity value at which the Ge layer becomes unstable and gradually detaches. Error bars represent the standard deviation of the average etching rate and porosity.

Supplementary Note. 5:
Electric Current Equations in Steady State

COMSOL Multiphysics provides interfaces for simulating static electric fields and currents [6]. When dealing with stationary electric currents in conductive materials, the stationary continuity equation must be considered. In a stationary system, the point form of Ohm's law states that:

$$J = \sigma E + J_e$$

where σ is the electrical conductivity (S/m), and Je is the externally generated current density (A/m²). The static form of the continuity equation then states:

$$\nabla . J = -\nabla . (\sigma \nabla V - J_e) = 0$$

To account for current sources, the equation can be generalized to:

$$-\nabla . (\sigma \nabla V - J_e) = Q_j$$

In 2D planar models, the Electric Currents interface assumes that the electric potential varies only along the *x* and *y* directions, remaining constant in the *z* direction. This means that the electric field (E) is tangential to the *xy*-plane. The interface solves the following equation, where *d* is the thickness in the *z* direction:

$$-\nabla . d(\sigma \nabla V - J_e) = dQ_j$$

In 2D axisymmetric models, the Electric Currents interface assumes axial symmetry for both the fields and geometry. In this case, the electric potential φ remains constant in the angular direction, implying that the electric field is tangential to the *rz*-plane.

Electrical Model

The Electric Currents physics interface was applied across all domains to simulate current flow within the Ge/Si system, utilizing two physics interfaces: Electric Currents and Electrostatics. These interfaces were employed to model the distribution of electric fields and current densities in a static (steady state) regime, with the objective of understanding the electrochemical etching behavior of Ge/Si under an applied electric potential. Table 1 summarizes the parameters defined for the domains. At the boundary, a prescribed mesh velocity in cylindrical coordinates was defined as follows:

$$\vartheta_r = -K_E . J_r$$

$$\vartheta_z = -K_E . J_z$$

Here, J_r and J_z represent the components of the current density vector in the r and z directions, respectively, and K is a constant that accounts for factors such as Ge and Si density and reaction valence in the electrical model.

The upper boundary of the HF electrolyte (cathode) was grounded, and an electrical potential of 1 V was applied to the lower boundary of the silicon substrate (anode). The structure's dimensions are shown in Fig.4, where the silicon at the interface, assumed to be highly doped, is labeled Si (a), and the silicon in the substrate is labeled Si (b). The material properties, such as conductivity and permittivity, were assumed to be constant and isotropic [7,8]. The simulation aimed to provide insights into the distribution of the electric field and current density, which are key factors in controlling the electrochemical etching process of Ge/Si heterostructure.

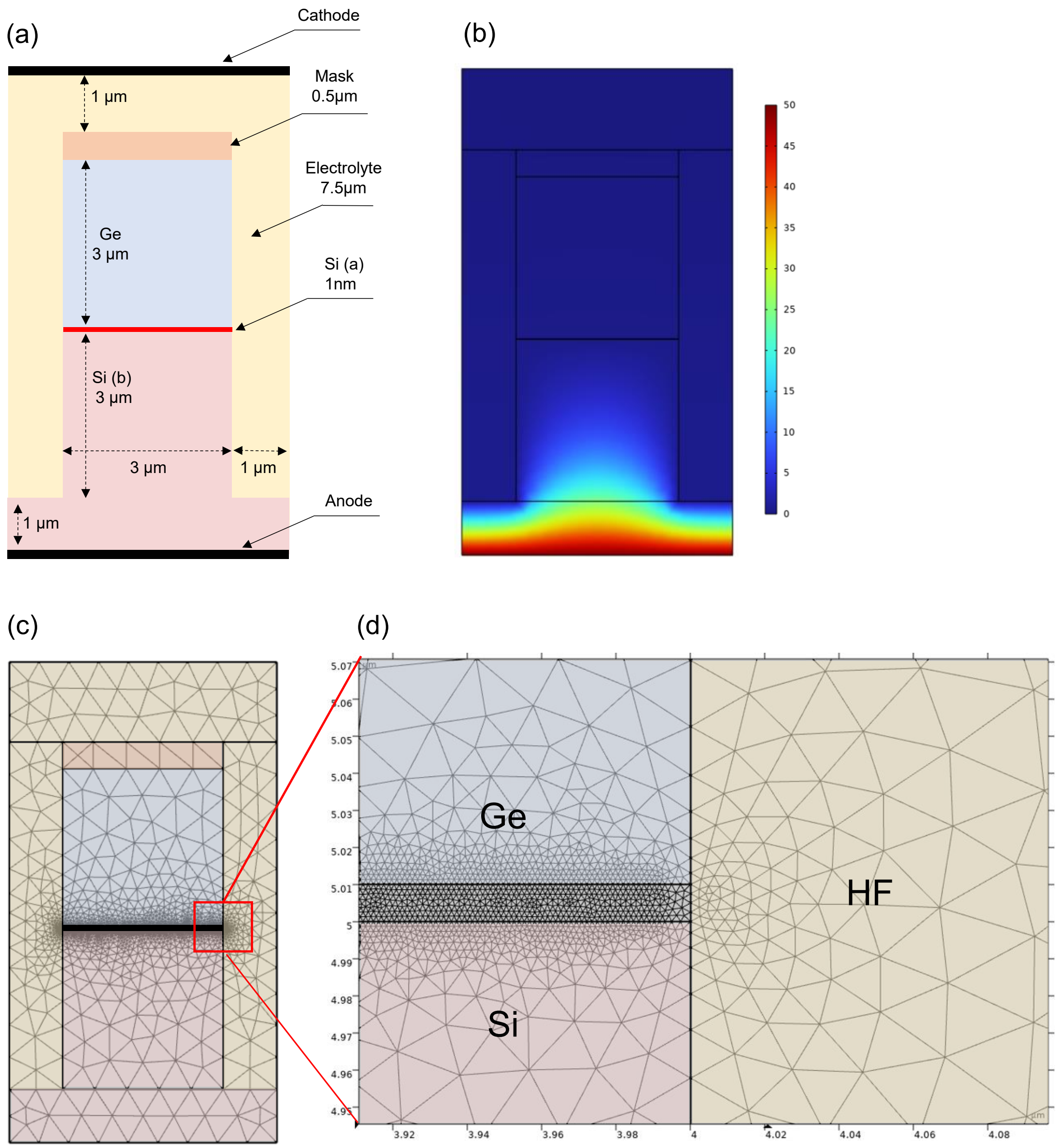

Fig. 4. (a) Scaled schematic of the model geometry. (b) Potential distribution map for the electrical model of the anodization process. (c) Triangular element mesh of the Ge/Si structure, incorporating the nanometric interface for precise simulation.

Table 1: Material properties of the domains in the electrical model

DOMAIN RELATIVE	ELECTRICAL CONDUCTIVITY	PERMITTIVITY
HF electrolyte	10^4 (S/m)	80.1
Mask	0 (S/m)	7.5
Ge	2 (S/m)	16
Si(a)	10^2 (S/m)	11.1
Si(b)	10 (S/m)	11.1

Supplementary Note. 6.

<u>Detailed Analysis of the Ge/Si Interface in the 2DFS Sample</u>

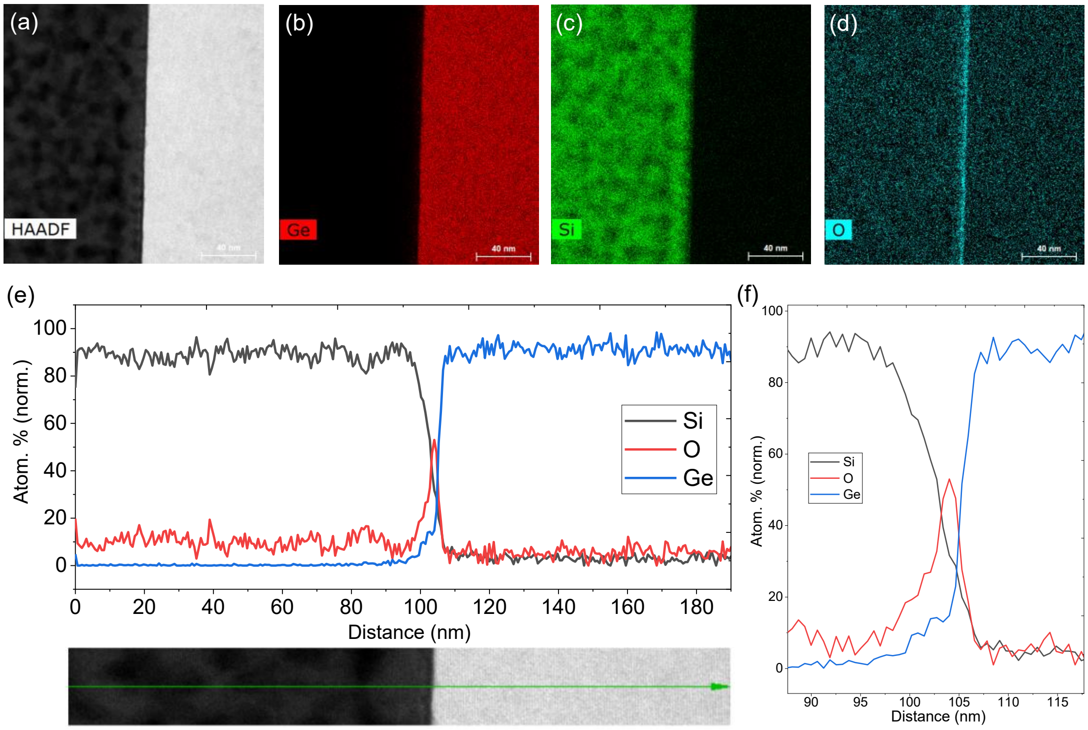

Fig. 5. Energy-dispersive X-ray spectroscopy (EDX) mapping of the Ge/Si interface in the 2DFS sample. (a) Cross-sectional scanning transmission electron microscopy (STEM) image and corresponding EDX element maps for (b) Si, (c) Ge, and (d) O. (e) EDX line profile along the horizontal green line and a zoomed-in view of the interface (f), showing the relative presence of oxygen within the nanometric interfacial layer, along with sharp variations in the Si and Ge signals. These observations are considered within the detection limits and resolution of the EDX technique.

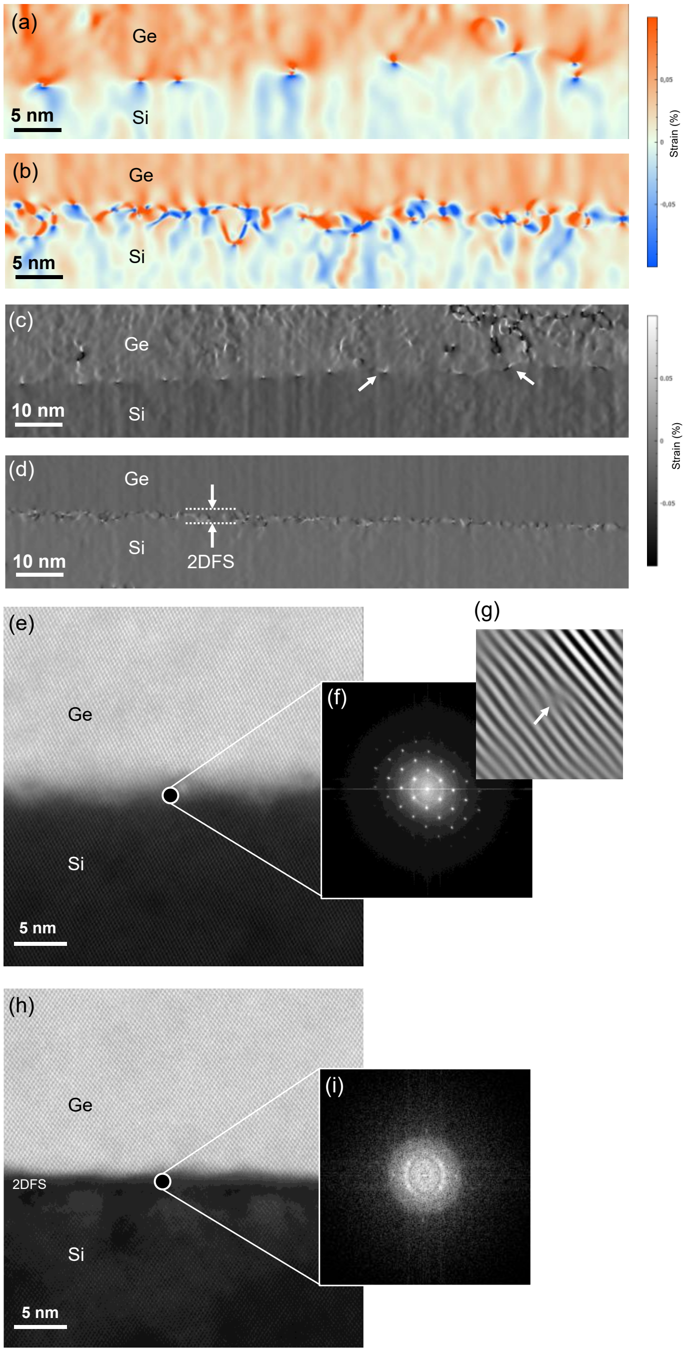

Fig. 6. Zoomed views of the strain component (ε_{xx}) for the Ge/Si reference (a) and 2DFS samples (b) highlight key differences. Misfit dislocations at the Ge/Si interface are analyzed in both the reference (c) and 2DFS samples (d), derived from the 2D strain component (ε_{xx}) using GPA. In the reference sample, the misfit dislocations appear as well-spaced dark points, whereas the 2DFS sample shows a disturbed region, suggesting suppression of these misfit dislocations. HR-STEM dark-field images of the Ge/Si interface are presented for the reference (e) and 2DFS sample (h). Corresponding FFT patterns in (f) and (i) confirm the crystallinity of the interface in the reference sample and reveal an amorphous signal in the 2DFS region. An inverse FFT (IFFT) image, obtained using the (11-1) spot, is displayed in (g), where lines in the numerical Moiré image align with lattice planes. At the interface, the edge component *b* of the 60° dislocation in Si is inclined by ~54° to the interface, with a single extra half-plane indicated by an arrow.

Supplementary Note. 7.
<u>Streamlining the Geometric Phase Analysis (GPA)</u>

The core principle of Geometric Phase Analysis (GPA) lies in comparing the phase of a set of ideal crystal planes (defined by a g-vector) with the phase of planes extracted from an image (defined by a mask at the corresponding g-vector) [9].

This approach can be understood by considering the image as being composed of a Fourier series, where the periodicity of the planes is analyzed in the frequency domain. Maps, and theory.

$$I(r) = \sum_{g} A_g e^{iP_g + 2\pi i g.r}$$

Where I represents the image intensity, r is the position within the image, and g denotes the periodicities (i.e., positions in reciprocal space). The terms A_g and P_g correspond to the amplitude and phase of the periodicity defined by g. By applying a simple mask, one (or a few) of these Fourier components can be selectively extracted by masking the FFT. Inverting the masked FFT then produces the complex image $H'_g(r)$. From this, the phase difference can be calculated as follows:

$$P_g(r) = Phase\left[H'_g(r)\right] - 2\pi g.r$$

In this expression, the first term represents the phase obtained from the masked FFT, while the second term corresponds to the phase calculated from the g-vector at which the FFT was masked. At this stage, the g-vector can be refined within a region of homogeneous strain. If the g-vector is slightly inaccurate, a gradient will appear in the phase of the uniform strain region. By fitting this gradient, the g-vector can be corrected using

$$\Delta g = \frac{1}{2\pi} \nabla P_g$$

Each phase can be used to determine the displacements along the direction of the lattice planes. To obtain the complete strain field, the phase must be calculated for two non-collinear g-vectors. The relationship between the phases and the displacement field, u, is given by:

$$\begin{pmatrix} P_{g1} \\ P_{g2} \end{pmatrix} = -2\pi \begin{pmatrix} g_{1x} & g_{1y} \\ g_{2x} & g_{2y} \end{pmatrix} \begin{pmatrix} u_x \\ u_y \end{pmatrix}$$

Here, g_{1x} and g_{1y} are the x and y components of the g-vector used to calculate the phase P_{g1}. By inverting this relationship, the displacements can be expressed in terms of the phases as follows:

$$\begin{pmatrix} u_x \\ u_y \end{pmatrix} = -\frac{1}{2\pi} \begin{pmatrix} a_{1x} & a_{1y} \\ a_{2x} & a_{2y} \end{pmatrix} \begin{pmatrix} P_{g1} \\ P_{g2} \end{pmatrix}$$

where it has been used:

$$\begin{pmatrix} g_{1x} & g_{1y} \\ g_{2x} & g_{2y} \end{pmatrix}^T = \begin{pmatrix} a_{1x} & a_{2x} \\ a_{1y} & a_{2y} \end{pmatrix}^{-1}$$

Finally, the distortion is determined by taking the derivative:

$$e = \begin{pmatrix} e_{xx} & e_{xy} \\ e_{yx} & e_{yy} \end{pmatrix} = \begin{pmatrix} {\delta u_x}/{\delta x} & {\delta u_x}/{\delta y} \\ {\delta u_y}/{\delta x} & {\delta u_y}/{\delta y} \end{pmatrix}$$

From this matrix, the strain (ε), rotation (ω), and dilation (Δ) can be readily calculated using:

$$\varepsilon = {1}/{2}\,(e + e^T)$$

$$\omega = {1}/{2}\,(e - e^T)$$

$$\Delta = Trace\,[e]$$

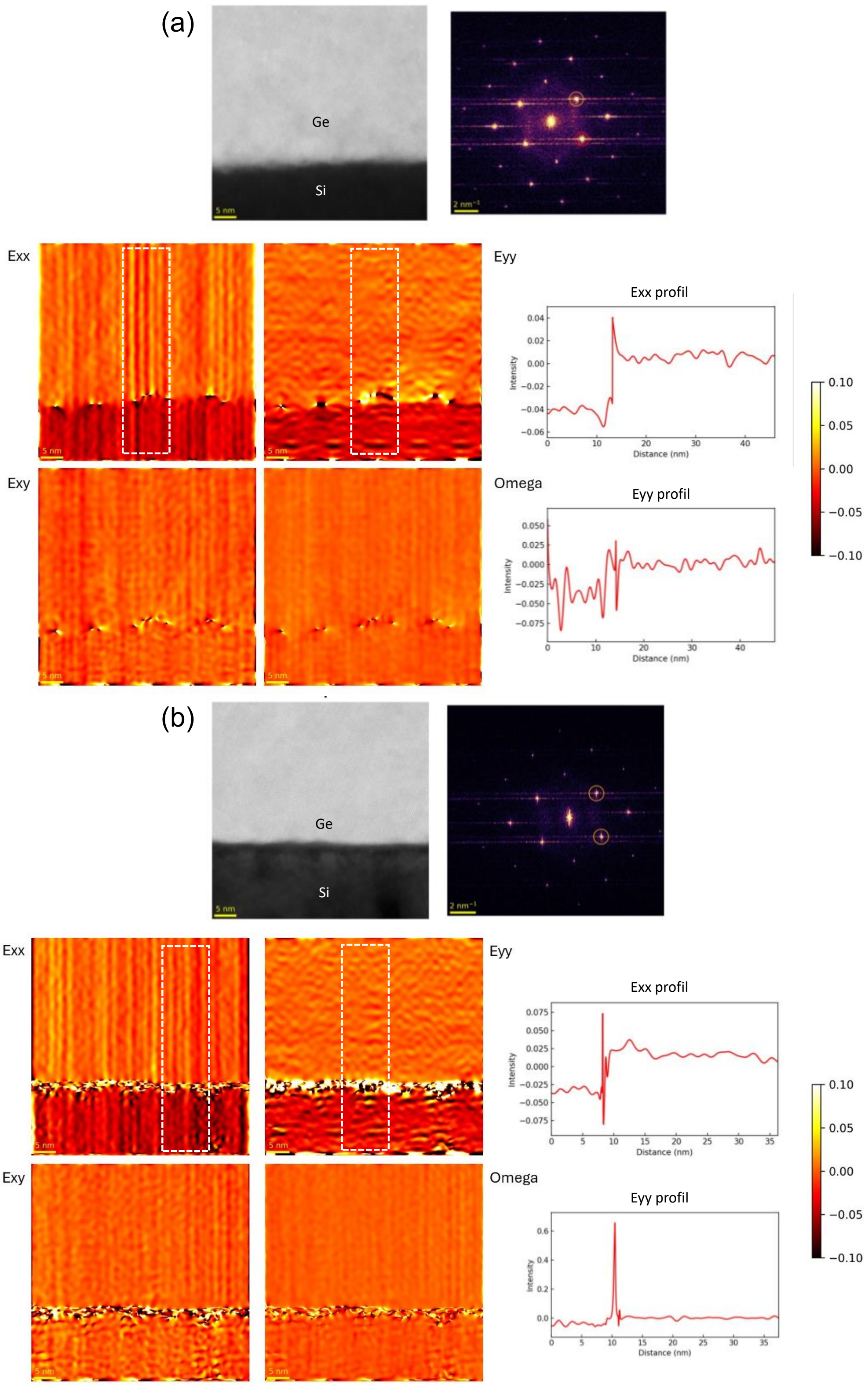

Fig. 7. High-magnification HRSTEM image and its FFT, along with 2D strain components (ε_{xx}, ε_{yy}, ε_{xy}), 2D rotation map (ω_{xy}), and in-plane strain profile extracted from a region defined by the dashed-line window at the Ge/Si interface. Strain analysis was performed using GPA for both the Ge/Si reference sample (a) and the 2DFS sample (b).

Supplementary Note. 8.

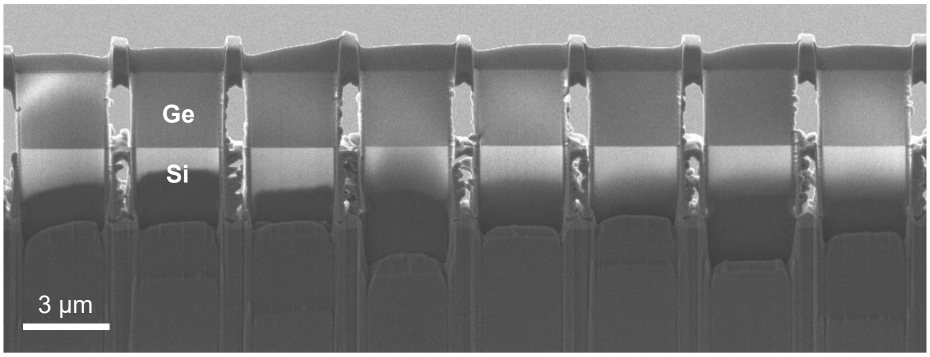

Fig. 8. A STEM image shows a lamella containing multiple Ge/Si towers fabricated from the same sample, enabling the analysis of effects across several towers. The TEM lamella was prepared using FIB milling through the following steps: a protective carbon cap was deposited in situ using a Pt deposition at 30 kV and 90 pA with a stage tilt of 52°, followed by milling of the target region with a Ga^+ ion beam at 30 kV and 2 nA. After milling, a tungsten probe was used to transfer the sample to a copper grid, welding the probe to the sample before severing the final connection. The sample was then attached to the copper grid by welding it to a post with carbon. Once the sample was confirmed to be securely bonded to the grid, the tungsten probe was detached. Finally, the sample was thinned to a thickness of 50–100 nm using a 2 kV Ga^+ ion beam and 30 pA.

Supplementary Note. 9.

Evaluation of Residual Defects

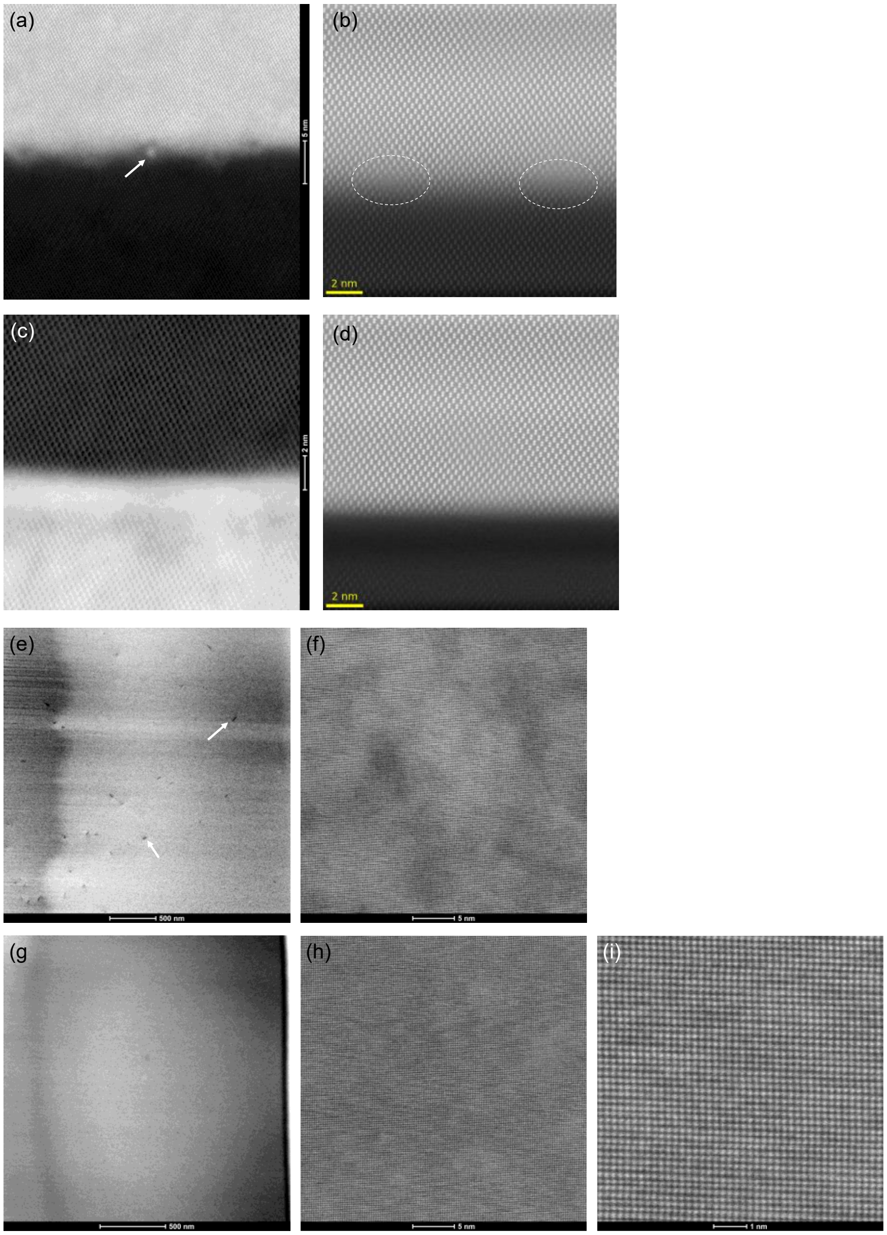

Fig. 9. (**a)** HAADF-STEM image of the Ge/Si reference sample. (**b)** High-resolution 4D-STEM strain map showing a dislocation core at the Ge/Si interface. **(c)** HAADF-STEM image of the Ge/Si 2DFS sample. **(d)** High-resolution 4D-STEM image of the Ge/Si 2DFS interface, revealing the absence of Ge–Si bonding across the 2D free surface. (**e)** Plan-view TEM micrograph of the Ge/Si reference sample showing multiple dislocation emergence sites. **(f)** High-magnification view of a dislocation emergence site from (**e),** imaged near the Ge surface**.** **(g)** Plan-view TEM image of the Ge/Si 2DFS sample, showing no observable dislocation emergence sites. **(h, i)** High-resolution plan-view TEM images of the Ge/Si 2DFS sample, highlighting atomic ordering in the top Ge layers.

Supplementary Note. 10.
<u>Wafer-scale uniformity</u>

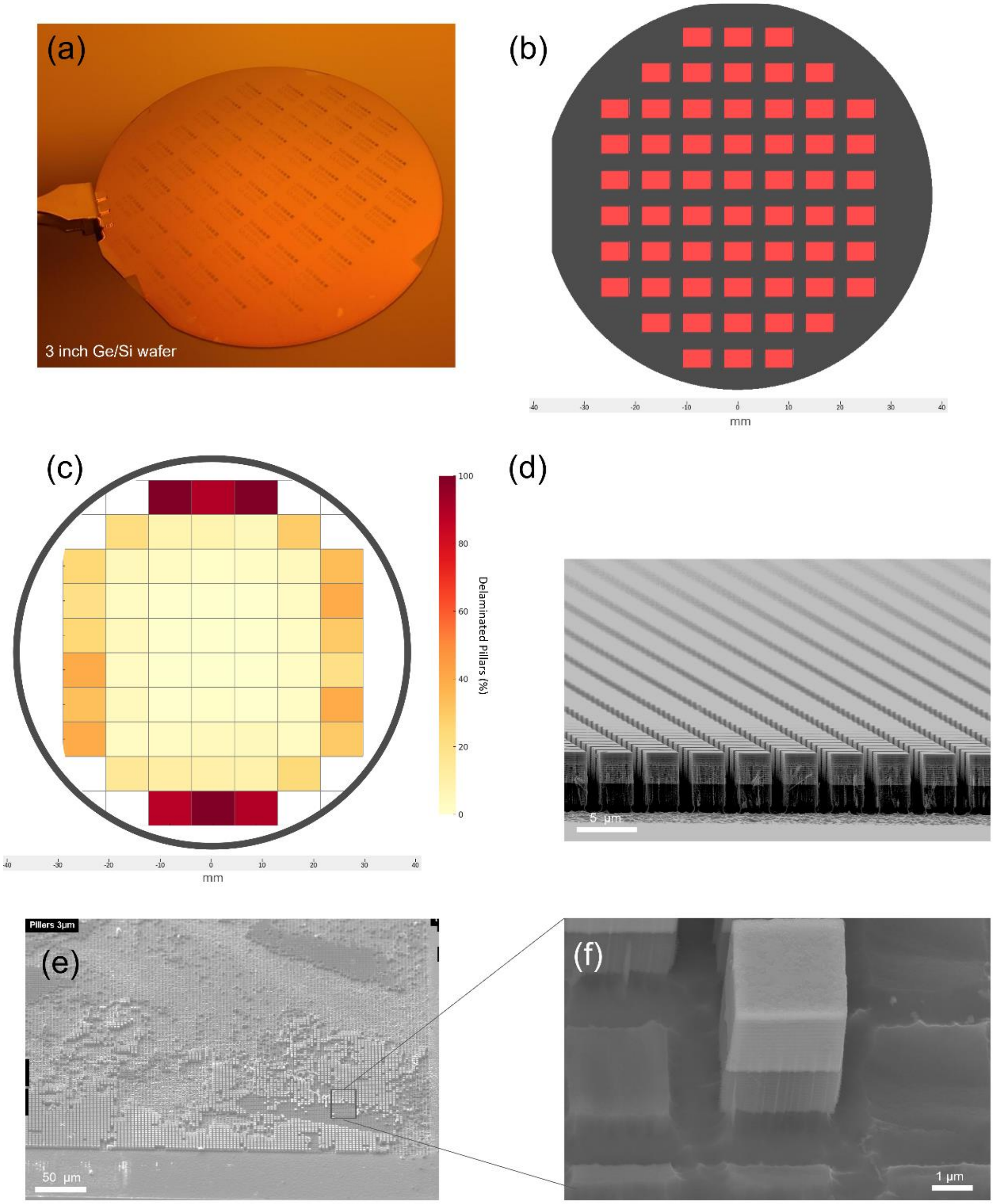

Fig. 10. Wafer-scale assessment of pillar stability following 2DFS processing. **(a)** Photograph of a 3-inch Ge/Si wafer patterned with micropillar arrays for 2DFS delamination. **(b)** Map indicating the positions sampled for assessment. **(c)** Heatmap showing the percentage of delaminated pillars at each location; lower values correspond to stable regions, while increased delamination is observed near the wafer edges. **(d)** SEM image of intact Ge/Si micropillars at the center of the wafer. **(e)** SEM overview from a wafer edge showing widespread pillar detachment. **(f)** High-magnification SEM image from the edge, indicating that fractures occur at the Si base due to local over-porosification.

Supplementary Note. 11.

Spreading Resistance Profiling Analysis

To probe the vertical electronic integrity of the Ge/Si heterostructure following electrochemical etching, we conducted spreading resistance profiling (SRP) on a beveled sample of 20 µm x 20 µm encompassing the full stack: Ge / 2DFS / porous-Si / Si substrate. The bevel angle (~9°) combined with a 2 µm lateral step size yielded an effective depth resolution of ~300 nm, enabling the resolution of each individual layer with high fidelity. The extracted SRP profile reveals a carrier concentration of ~10^{15} cm^{-3} within the Ge layer, consistent with the intended doping level and indicating that the etching process does not perturb the Ge carrier profile. A lower apparent carrier density is observed in the porous Si region (~10^{16} cm^{-3}), reflecting the increased resistivity associated with the nanostructured matrix. However, this value may not correspond to the actual doping concentration, as current redistribution effects and percolation limitations within the porous network can distort the local measurement. The signal eventually recovers to ~10^{18} cm^{-3} in the underlying crystalline Si, in good agreement with the nominal substrate doping.

Taken together, these results confirm that the electrochemical etching process preserves the resistive uniformity of the Ge layer and does not introduce substantial carrier trapping or mobility-limiting defects at the interface. The data support the structural and electronic resilience of the stack under process-induced porosification.

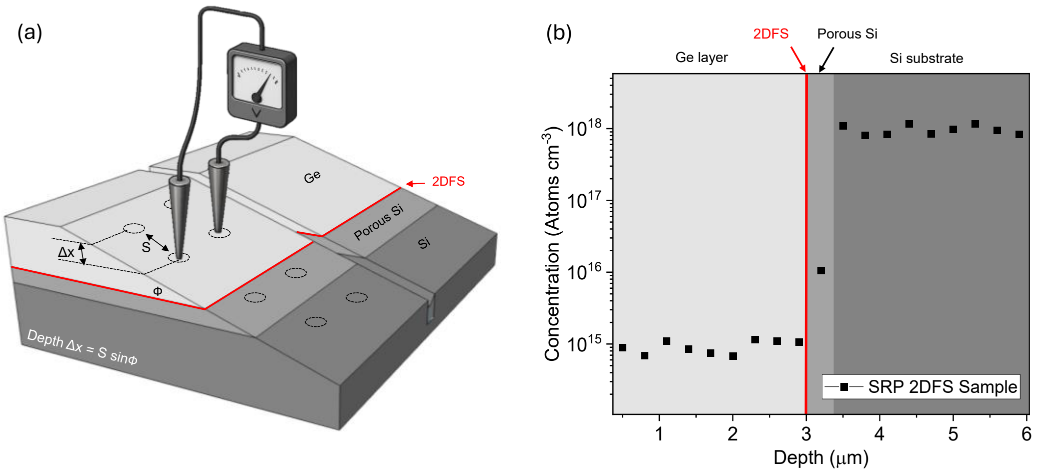

Fig. 11. (a) Schematic illustration of the spreading resistance profiling (SRP) measurement performed on a Ge/Si pillar structure (20 µm × 20 µm), following bevel preparation. **(b)** Extracted carrier concentration profile as a function of depth, revealing the distinct Ge, porous Si, and bulk Si regions.

Supplementary Note. 12.

Residual Stress Assessment and Stability under Thermal Cycling

To assess residual strain in the Ge epilayer and its implications for long-term mechanical and electronic stability, micro-Raman spectroscopy was conducted on three representative samples: (i) bulk Ge reference, (ii) 3 × 3 μm² Ge/Si micropillars without interface modification, and (iii) 3 × 3 μm² Ge/Si micropillars incorporating a 2DFS interface. Measurements were performed using a 532 nm excitation in backscattering geometry. With a probing depth <20 nm in Ge [10], the Raman signal is surface-sensitive, offering an upper estimate of in-plane residual strain. Figure S2 presents the normalized Raman spectra for the three configurations. The Ge phonon peak appears at 300.84 cm^{-1} in bulk Ge. A downshift to 300.40 cm^{-1} is observed in the Ge/Si micropillars, corresponding to a tensile strain of ~0.10%. The 2DFS-modified sample shows a peak at 300.65 cm^{-1}, indicating a reduced strain of ~0.05%. These values were extracted using $\Delta\nu = -b \cdot \varepsilon$, with $b = 415\ cm^{-1}$ [11]. Phonon linewidths also reflect microstructural differences: the FWHM increases from 1.30 cm^{-1} (bulk Ge) to 1.92 cm^{-1} (Ge/Si) but narrows to 1.63 cm^{-1} in the 2DFS sample, confirming improved crystalline uniformity and reduced microstrain. The spectra show a single dominant Raman mode in all cases, consistent with high-quality single-crystal Ge in (001) orientation [12]. These results demonstrate that the 2DFS interface significantly alleviates substrate-induced residual stress in the Ge film, thereby supporting its structural robustness and long-term stability.

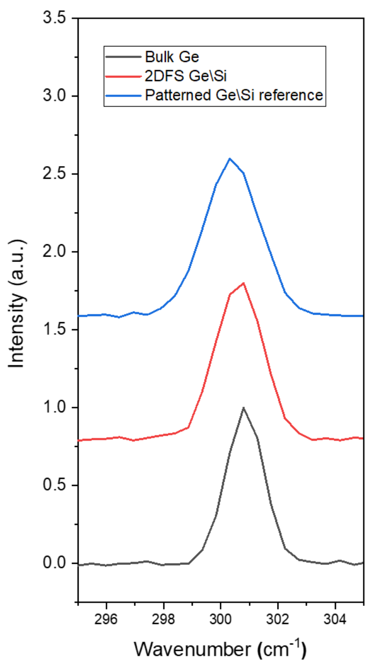

Fig. 12. Raman spectroscopy analysis of residual strain in Ge/Si structures. (a) Normalized Raman spectra for three representative samples: bulk Ge (black), Ge/Si micropillars without interface engineering (blue), and Ge/Si micropillars incorporating the 2D free-surface (2DFS) interface (red). (b) Extracted peak positions and full widths at half maximum (FWHM) highlight the reduction in tensile strain and microstrain in the 2DFS sample compared to the conventional Ge/Si structure. The peak position in bulk Ge (300.84 cm^{-1}) serves as an unstrained reference. These results demonstrate that the 2DFS interface effectively mitigates substrate-induced residual stress in the Ge layer.

Thermal Cycling Test Protocol and Results

To assess the thermal stability of the 2DFS integrated Ge/Si micropillars, cyclic annealing tests were performed by subjecting the samples to 30 thermal cycles between 25 °C and 400 °C in a forming gas atmosphere (H_2 10% / N_2 90%) to prevent oxidation. The temperature ramp rate was approximately 10 °C/min, with a dwell time of 10 minutes at each temperature extreme to allow thermal equilibration. Micro-Raman spectroscopy was conducted at a fixed location after each cycle to monitor changes in the Ge optical phonon mode, which is sensitive to strain and lattice quality. The Raman peak position remained constant at 300.65 cm^{-1} throughout the test, while the full width at half maximum (FWHM) showed only minor fluctuations (~±0.03 cm^{-1}), indicating no accumulation of microstrain or degradation of the crystal lattice. Visual inspection after the thermal cycling revealed no cracks or other signs of mechanical damage, underscoring the structural integrity provided by the 2DFS architecture. Although this test is shorter than typical industrial reliability protocols—which often require hundreds to thousands of cycles—it offers meaningful preliminary evidence that the 2DFS design effectively mitigates thermal mismatch stresses and maintains mechanical stability under repeated thermal cycling.

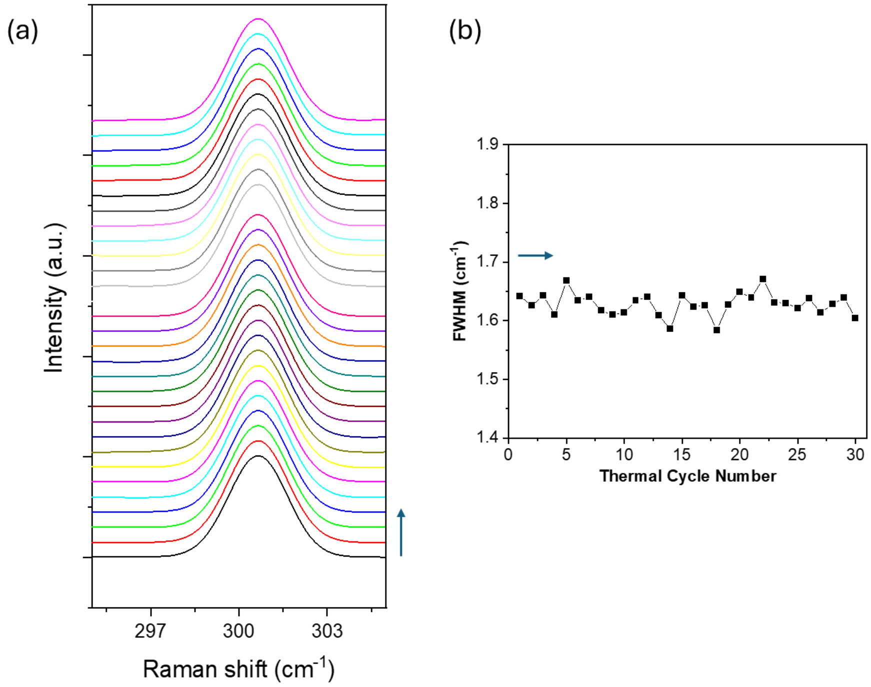

Fig. 13. Thermal cycling stability of the 2DFS integrated Ge/Si micropillars. **(a)** Raman spectra measured at the same location after each of the 30 thermal cycles between 25 °C and 400 °C in forming gas (H_2 10% / N_2 90%). The Ge optical phonon peak remains stable at around 300.6 cm^{-1} with no significant shift or broadening observed, indicating preserved strain-relaxed lattice. **(b)** Evolution of the Raman full width at half maximum (FWHM) across the cycles, showing only minor fluctuations (~±0.03 cm^{-1}), confirming absence of thermally induced microstrain or lattice degradation. Post-test inspection revealed no cracks or visible damage, supporting the excellent mechanical stability of the 2DFS architecture under thermal cycling.

References :

1. Darnon, M. *et al.* Deep germanium etching using time multiplexed plasma etching. *Journal of Vacuum Science & Technology B, Nanotechnology and Microelectronics: Materials, Processing, Measurement, and Phenomena* **33**, 0–7 (2015).
2. Bioud, Y. A. *et al.* Fast growth synthesis of mesoporous germanium films by high frequency bipolar electrochemical etching. *Electrochim Acta* **232**, 422–430 (2017).
3. Memming, R. & Schwandt, G. *Anodic Dissolution of Silicon in Hydrofluoric Acid Solutions*. *SURFACE SCIENCE* vol. 4 (1966).
4. Looyenga, H. Dielectric Constants of Heterogeneous Mixtures. *Physica* **31**, 401–06 (1965).
5. Canham, L. *Properties of Porous Silicon*. (1997).
6. *COMSOL Multiphysics Reference Manual*. www.comsol.com/blogs (1998).
7. Ivanov, A. & Ivanov, A. *Simulation of Electrochemical Etching of Silicon with COMSOL*. https://www.researchgate.net/publication/264118364.
8. Ivanov, A., Mescheder, U. & Ivanov, A. *Dynamic Simulation of Electrochemical Etching of Silicon with COMSOL Dynamic Simulation of Electrochemical Etching of Silicon*. https://www.researchgate.net/publication/264118212.
9. M J Hytch, Snoeck, E. & Kilaas, R. *Quantitative Measurement of Displacement and Strain Fields from HREM Micrographs*. *Ultramicroscopy* vol. 74 (1998).
10. H. J. Yvon, Strain measurements of a Si cap layer deposited on a SiGe substrate determination of Ge content. www.horiba.com, 2013.
11. Y. Y. Fang, J. Tolle, R. Roucka, A. V. G. Chizmeshya, J. Kouvetakis, V. R. D'Costa, and J. Menendez, Appl. Phys. Lett. 90, 061915 (2007).
12. I. H. Campbell and P. M. Fauchet, Solid State Commun. 58, 739 (1986).
